# Validation of Shared and Specific Independent Component Analysis (SSICA) for Between-Group Comparisons in fMRI

**DOI:** 10.3389/fnins.2016.00417

**Published:** 2016-09-27

**Authors:** Mona Maneshi, Shahabeddin Vahdat, Jean Gotman, Christophe Grova

**Affiliations:** ^1^Montreal Neurological Institute and Hospital, McGill UniversityMontreal, QC, Canada; ^2^Multimodal Functional Imaging Laboratory, Biomedical Engineering Department, McGill UniversityMontreal, QC, Canada; ^3^Functional Neuroimaging Unit, Centre de Recherches de l'Institut Universitaire de Gériatrie de Montréal, Université de MontréalQC, Canada; ^4^Psychology Department, McGill UniversityMontreal, QC, Canada; ^5^PERFORM Centre and Physics Department, Concordia UniversityMontreal, QC, Canada

**Keywords:** functional connectivity (FC), independent component analysis (ICA), between-groups comparison, fMRI, statistical modeling

## Abstract

Independent component analysis (ICA) has been widely used to study functional magnetic resonance imaging (fMRI) connectivity. However, the application of ICA in multi-group designs is not straightforward. We have recently developed a new method named “shared and specific independent component analysis” (SSICA) to perform between-group comparisons in the ICA framework. SSICA is sensitive to extract those components which represent a significant difference in functional connectivity between groups or conditions, i.e., components that could be considered “specific” for a group or condition. Here, we investigated the performance of SSICA on realistic simulations, and task fMRI data and compared the results with one of the state-of-the-art group ICA approaches to infer between-group differences. We examined SSICA robustness with respect to the number of allowable extracted specific components and between-group orthogonality assumptions. Furthermore, we proposed a modified formulation of the back-reconstruction method to generate group-level *t*-statistics maps based on SSICA results. We also evaluated the consistency and specificity of the extracted specific components by SSICA. The results on realistic simulated and real fMRI data showed that SSICA outperforms the regular group ICA approach in terms of reconstruction and classification performance. We demonstrated that SSICA is a powerful data-driven approach to detect patterns of differences in functional connectivity across groups/conditions, particularly in model-free designs such as resting-state fMRI. Our findings in task fMRI show that SSICA confirms results of the general linear model (GLM) analysis and when combined with clustering analysis, it complements GLM findings by providing additional information regarding the reliability and specificity of networks.

## Introduction

In the context of functional magnetic resonance imaging (fMRI), the temporal and spatial structure of blood oxygenation level–dependent (BOLD) signal make Independent Component Analysis (ICA) a suitable method to study changes in brain networks between different clinical populations or following interventions which induce neural plasticity (Fox and Raichle, 2007; Albert et al., 2009; Calhoun et al., 2009; Assaf et al., 2010; Vahdat et al., 2014).

In particular, the analysis of resting state data, with no temporal constraint on the experimental design, underscores the application of an exploratory multivariate method such as ICA. Although ICA has been successfully applied to a single homogeneous group of subjects (Calhoun et al., 2001; Beckmann et al., 2005; Esposito et al., 2005), the comparison of individual extracted networks between different experimental groups or conditions is not a trivial task, which makes the subsequent statistical inferences quite challenging (Calhoun et al., 2009; Sui et al., 2009b; Vahdat et al., 2012).

Generally speaking, there are two ways of performing group level ICA (gICA), none of them being fully appropriate for between-group comparisons. One way is to aggregate the data from all subjects and all groups in one single matrix, and then to apply ICA irrespective of group membership information (Assaf et al., 2010; Ma et al., 2011). Theoretically, the extracted independent components (ICs) from this approach account for the variability from all individuals in the dataset, so they tend to represent those networks that are common to all groups. We will refer to networks which are common across all groups of subjects as shared components. Based on this concatenation approach, some methods, such as back-reconstruction (Calhoun et al., 2001) and dual regression (Filippini et al., 2009) have been proposed in order to dissociate the contribution of each subject of each group within gICA results, thus allowing statistical comparisons.

However, these methods are still left with the problem of gICA blind concatenation, which is the lack of access to group membership information at the component-extraction level. Consequently, it results in reduced sensitivity to extract between-group differences at the first level of the analysis. Another gICA approach consists of concatenating individuals from each group separately and then applying ICA on the concatenated matrices of each group (Calhoun et al., 2001; Albert et al., 2009). As multiple ICA runs result in various decomposition patterns across groups, the comparison between components with different spatial characteristics is then quite difficult, and making valid statistical inferences even more challenging. For example, if a network is present among the extracted components in one group and not in the other, it might be simply due to insufficient number of extracted components in one of the groups. Furthermore, comparing the power of two components, which are comprised of similar brain voxels, is not statistically correct, unless the weighting coefficients across the voxels in the two spatial maps are similar.

Several methods have been proposed to overcome the challenge of between-group comparisons using ICA (Guo and Pagnoni, 2008; Sui et al., 2009a,b) or local linear discriminant analysis (McKeown et al., 2007; Palmer et al., 2010). Generally, these studies were able to increase sensitivity to detect group differences by extracting a set of features from the datasets, and maximizing the separability of the extracted features between groups. As such, these methods operate in the feature space and cannot be applied directly on the original time series of BOLD data. This may limit their application in cases such as resting-state analysis where task timing information is not available, and feature extraction in time domain is challenging. Tensorial ICA proposed by Beckmann and Smith (2005) is another group-level ICA approach which provides a systematic and robust way to compare results of ICA across different subjects by decomposition of data in terms of their temporal, spatial, and subject-dependent variations. However, in temporal domain, tensorial ICA can only be applied to fMRI activation paradigms, where a common temporal activation profile across subjects is provided by the task, although its application on resting-state data has been proposed by performing group ICA model on voxel-wise power spectra profiles (Damoiseaux et al., 2008).

Furthermore, a unified framework for performing gICA has been proposed (Guo and Pagnoni, 2008). The group structure proposed by tensorial ICA (Beckmann and Smith, 2005) and the GIFT software (Calhoun et al., 2001) can be formulated using this general framework (Guo and Pagnoni, 2008). A group tensor model was proposed in this study, in which the representative mixing matrix time courses are allowed to be different across groups. This group model is a natural extension of tensorial ICA for multi-group data, but is more restricted in terms of the subjects' mixing matrices than the GIFT software. In another study, Lukic et al. (2002) used time-delayed autocorrelations to obtain independent signal components in a multi-group fMRI design. Other gICA approaches include FENICA (Schopf et al., 2010), which uses correlation coefficients between components to extract spatially consistent networks across a group of subjects, and CanICA (Varoquaux et al., 2010) that employs generalized canonical correlation analysis to identify a subspace of reproducible components in a group of subjects.

Other methods using clustering algorithms and multi-level PCA have been proposed to group individual-level components into a set of stable clusters (Esposito et al., 2005; Zhang et al., 2010; Hyvarinen, 2011; Boly et al., 2012; Yang et al., 2012; Hyvarinen and Ramkumar, 2013). Recently, Hyvarinen and Ramkumar (2013) proposed a statistical approach based on inter-subject or inter-session consistency of ICA components to estimate the significance of group-level spatial maps. Other researchers have proposed to use hierarchical clustering methods on subject and group level component decomposition combined with Bootstrap resampling techniques to extract stable and reliable group level networks (Bellec et al., 2010; Boly et al., 2012). The implementation of such approaches in between-group designs has yet to be investigated.

We proposed a new algorithm, called Shared and Specific Independent Component Analysis (SSICA), to address the aforementioned limitations of the regular ICA approaches in the context of multi-groups/factorial designs (Vahdat et al., 2012). SSICA is based on adding a new constraint to the cost function of FastICA algorithm, originally proposed by Hyvarinen and Oja (2000), to simultaneously deal with the data of multiple groups within a single ICA decomposition. It extracts and concurrently classifies ICs as being either shared across groups or specific to one or a subset of groups (referred to as specific components). The specific components represent the pattern of differences between groups or conditions that, generally, are of interest in neuroimaging studies. Basically, in order to extract specific components related to one group (called the “matching” group), a new cost function was defined to minimize their projection magnitude on the data of the other groups (called the “opposite” groups). Therefore, both independence and orthogonality principles, respectively at the component extraction level and at the group level, are considered simultaneously in the SSICA optimization. The convergence of SSICA algorithm is proved to be linear (Appendix B in Vahdat et al., 2012; Supplementary Material), which suggests that both the independence maximization and the orthogonality constraint imposed by a regularization term in the SSICA's cost function can be achieved with sufficient number of iterations. However, it is noteworthy that if the orthogonality assumption cannot be met through several iterations, the algorithm only optimizes the independence criterion by reducing the number of specific components to zero; i.e., only the “maximum” desired number of specific components can be set in SSICA, and the actual number of extracted ones depends on the convergence criteria.

The philosophy behind SSICA is that it allows one to systematically distinguish between changes in which the structure of activated network is different across groups (specific components), from those in which the structure of network is similar but the network power is different across groups (i.e., shared components with different time course's power). The change in the network structure between groups can be caused by adding (or removing) areas from an existing network, or even by recruiting the same areas but with different weightings across activated voxels: all likely situations in functional brain studies (Vahdat et al., 2014). Furthermore, the fact that SSICA tries to minimize the projection magnitude of the specific components does not mean that they have zero magnitude in the “opposite” group. But, when the convergence is achieved, it guaranties that the magnitude of specific components is significantly lower in the “opposite” than in the “matching” group. On the other hand, from a theoretical point of view, because of the blind concatenation of data across groups with no further information regarding group membership, regular gICA approach is probably more efficient in detecting power differences across shared components; although, in sufficiently powered experiments, the application of back-reconstruction or dual regression methods can be particularly useful to uncover potential differences in the spatial maps of the extracted components across groups/conditions (Calhoun et al., 2001).

The performance of SSICA has been investigated in Vahdat et al. (2012), using one-dimensional and two-dimensional simulated fMRI-like dataset using SimTB software (Erhardt et al., 2012). In the current study, we aim to test the validity of the SSICA method on real fMRI datasets, therefore evaluating for the first time the performance of SSICA on high dimensionality fMRI datasets. To do so, we collected two sets of fMRI data: (I) one during resting-state periods based on which we generated realistic simulations (hybrid fMRI) composed of arbitrary focal activations (patches) added to real resting-state fMRI data, (II) the other during a finger-tapping experiment with either visual or auditory cue. Having finger-tapping cued with auditory and visual stimuli allowed us to create two conditions, in which either the auditory and sensorimotor areas or the visual and sensorimotor areas are mostly activated. Using such paradigm, our hypothesis is that the sensorimotor network should play the role of a shared component, while the auditory and visual networks participate as specific components, each preferentially activated in one of the conditions. Adding patches of activation to the resting-state fMRI dataset (hybrid fMRI data) has been previously employed by Calhoun et al. (2001) and by our group (Dansereau et al., 2014). Even though patches might not perfectly mimic the actual resting-state networks, they make it possible to compare performance of different methods on the resting-state fMRI dataset by giving access to a controlled reference.

To adapt SSICA to the high dimensionality of fMRI data, we propose some modifications to our original method by improving the initialization of the iterative algorithm. In addition, we adapted the principle of back-reconstruction method proposed in Calhoun et al. (2001), in order to apply it to the results of SSICA. Back-reconstruction allows projecting group-level ICA results at the single subject level, thus providing an efficient way to generate group-level *t*-statistics maps from the extracted ICs.

Several simulations are provided to investigate characteristics of SSICA where the number of networks specific to each condition is not correctly set, as could occur in real circumstances because of the high degree of variability in fMRI brain network analysis. Also, we examined the properties of SSICA in the presence of “so-called” partially-specific components, where the specific networks' orthogonally assumptions are not fully met. The results of these simulations, as well as the results of SSICA on the acquired finger-tapping data are compared with the time-concatenation gICA method, implemented in the GIFT software (Calhoun et al., 2001), http://mialab.mrn.org/software/gift. For the finger-tapping task, we further evaluated the consistency and the specificity of SSICA results, following the methodology we previously described in Maneshi et al. (2014).

## Methods

### Standard ICA and group-level ICA

In linear ICA, an observed *T*×*M* matrix of random variables *Y* is decomposed based on the following generative model:

(1) $$\begin{array}{l} Y=AS \end{array}$$

Where *S* is an *N* × *M* dimensional matrix (*N* < *T*) whose rows are mutually independent (sources), and *A* is a *T* × *N* mixing matrix. Each row of matrix *S* and its corresponding column in the mixing matrix constitute a single component or network. In the context of fMRI connectivity analysis, extracting spatially independent components (spatial ICA) is usually preferred to temporally ICs, which implies setting *T* to the number of acquired volumes in time and *M* to the number of voxels. As a preprocessing step in gICA fMRI analysis, usually two levels of principal component analysis (PCA) are performed, one on the data of each individual and another one on the concatenated data of all individuals (Calhoun et al., 2009), although more recent studies have suggested a three-step PCA data reduction approach, similar to the one used in SSICA, for multi-group fMRI data (Zhang et al., 2010). Let *n*_1_ be the number of subjects in group-1, and *n*_2_ the number of subjects in group-2. Assume $Y_{i}^{j}$ denotes the $T_{i}^{j}$ × *M* zero-mean data of subject *i* in group *j*, where $T_{i}^{j}$ is the number of acquired volumes for that subject, assuming that all the subjects were acquired with the same spatial matrix (M voxels after co-registration). The first level PCA reduces the dimensions of the data of each subject in group *j* from $T_{i}^{j}$ to *T*_*j*_, using the following projection $F_{i}^{j}$: $X_{i}^{j}=F_{i}^{j}Y_{i}^{j}$.

In the spatial ICA framework, temporal concatenation of data of different subjects in one single matrix is commonly used as a method to perform gICA (Calhoun et al., 2009):

(2) $$\begin{array}{rcl} {\tilde{X}}_{1} & = & \left[\begin{array}{c} X_{1}^{1}\\ .\\ .\\ .\\ X_{n_{1}}^{1} \end{array}\right];{\tilde{X}}_{2}=\left[\begin{array}{c} X_{1}^{2}\\ .\\ .\\ .\\ X_{n_{2}}^{2} \end{array}\right]\\ \left[\begin{array}{c} {\tilde{X}}_{1}\\ {\tilde{X}}_{2} \end{array}\right] & = & AS \end{array}$$

Where ${\tilde{X}}_{1}$ and ${\tilde{X}}_{2}$ are the concatenated reduced data of group-1 and group-2, respectively. A second level PCA data reduction is then necessary to make the mixing matrix square, reducing the dimension of the concatenated data from *T*_1_*n*_1_ + *T*_2_*n*_2_ to N using the following projection: $\tilde{X}=\tilde{G}\left[\begin{array}{c} {\tilde{X}}_{1}\\ {\tilde{X}}_{2} \end{array}\right]$, where N is the desired number of extracted components. As described earlier, the gICA approach that concatenates data from subjects of both groups in a single matrix (Equation 2) is preferable to performing separate ICA on the data of each group. Hence, in this paper, we limited the comparison of the SSICA to this gICA strategy and we used GIFT implementation of this gICA strategy.

### SSICA model

**Figure 1:**
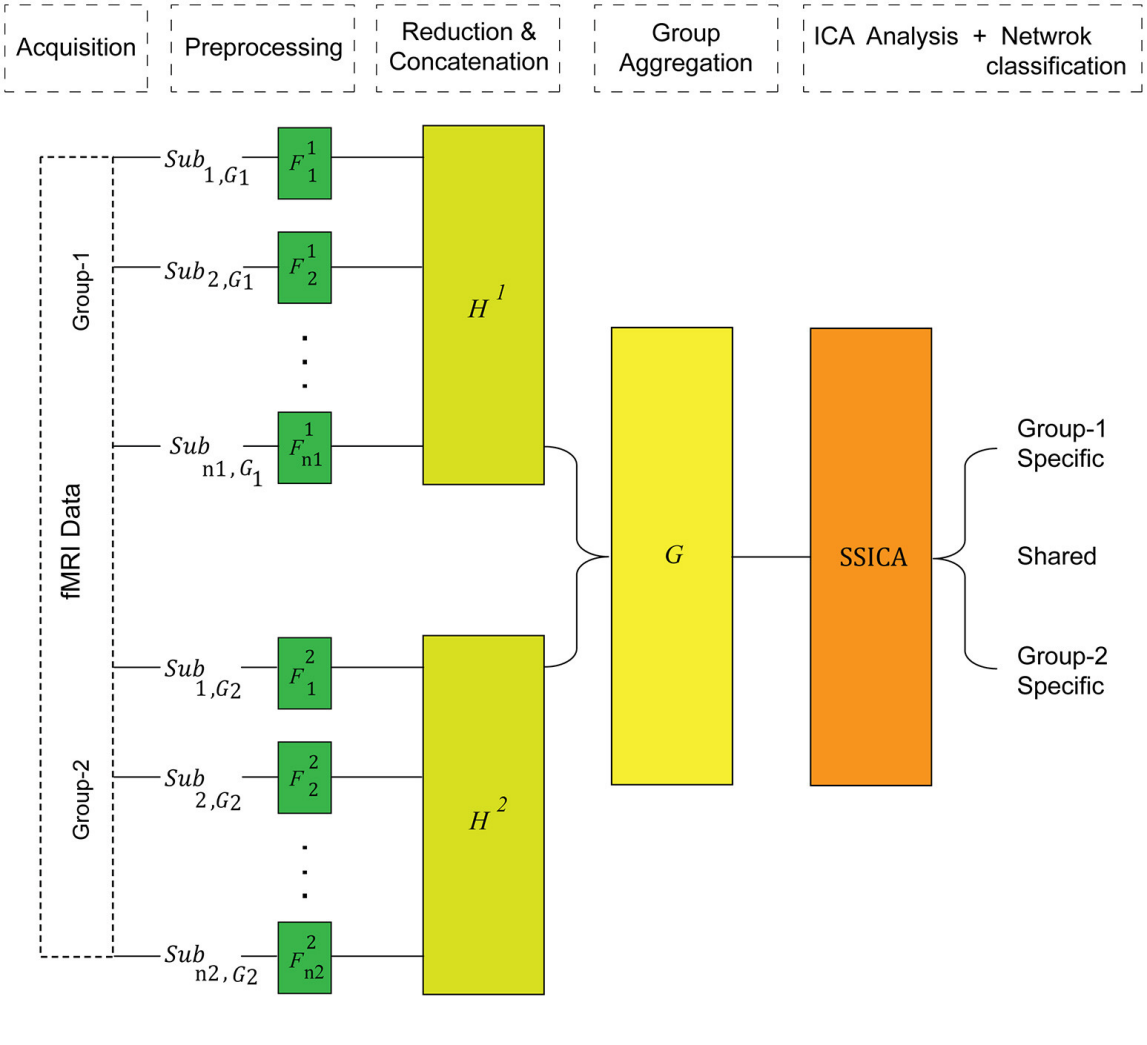
**The SSICA algorithm schematic**. There are three levels of data whitening and dimension reduction in SSICA. *F, H*, and *G* represent the projection matrices at the first (subject), second (within-group), and the third (between-group) levels of data reduction, respectively.

In contrast to the regular gICA, SSICA employs a 3-step PCA data reduction and whitening procedure on, (1) each individual data, (2) each group's concatenated data, and (3) multi-group aggregate data, as illustrated in Figure 1. Although the first data reduction step is not necessary for the proper operation of SSICA, it is recommended in fMRI analysis due to the computational burden. The subject-level reduction step uses the same projection matrix, $F_{i}^{j}$ described above.

At the second level, the temporally concatenated data of all subjects of each group is whitened and PCA-reduced using the following equation: $X_{j}=H^{j}{\tilde{X}}_{j},j=1,2$ where *H*^1^ is an *N*_*g*1_ × *n*_1_*T*_1_ whitening matrix for group-1, and *H*^2^ is an *N*_*g*2_ × *n*_2_*T*_2_ whitening matrix for group-2. Here, *X*_1_ denotes the whitened concatenated observed data matrix of group-1, and *X*_2_ denotes the whitened concatenated data from group-2. As PCA extracts directions of maximum variance in the observed data, the order of subjects in each group does not change the extraction results.

At the third level, group data are aggregated row-wise, and further whitened and PCA-reduced using an *N* × (*N*_*g*1_ + *N*_*g*2_) projection matrix, *G* : *X* = *G*$\left[\begin{array}{c} X_{1}\\ X_{2} \end{array}\right]$. Here, *N* defines the number of ICs that will be extracted by ICA. Let us assume that group-1 and group-2 data can be reconstructed by *K*_*g*1_ and *K*_*g*2_ ICs, respectively (true number of components in each group). Assuming *K* shared components among them ($s_{i}^{sh},i=1,..,K$), we can then decompose the generative model given in Equation (1) into two parts for each group: components shared between the two groups ($s_{i}^{sh}$), and components specific to each group ($s_{i}^{sp,1}\;or\;s_{i}^{sp,2}$):

(3) $$\left[\begin{array}{c} X_{1}\\ X_{2} \end{array}\right]=\left[\begin{array}{ccc} A_{1}^{\;sh} & A_{1}^{\;sp} & 0\\ A_{2}^{\;sh} & 0 & A_{2}^{\;sp} \end{array}\right]\left[\begin{array}{c} S^{sh}\\ S_{1}^{\;sp}\\ S_{2}^{\;sp} \end{array}\right]$$

where *s*^*sh*^ and *s*^*sp, j*^ are columns of ${S_{j}}^{T},j=1,2$ arranged according to the shared and specific labeling, and *a*^*sh, j*^, *a*^*sp, j*^ are the corresponding columns in the mixing matrices $A_{j}^{sh}$ and $A_{j}^{sp}$. Here, *K*_1_ = *K*_*g*1_ − *K* is the true number of specific components of group-1 ($s_{i}^{sp,1},i=1,..,K_{1}$), and *K*_2_ = *K*_*g*2_ − *K* is the true number of specific components of group-2 ($s_{i}^{sp,2},i=1,..,K_{2}$). Note that, the reduced dimension of aggregate data specified at the third level PCA, N, should be set greater than or equal to the total number of components in both groups (i.e., *N* ≥ *K*_1_ + *K*_2_ + *K*). Also, in SSICA the maximum number of specific components that can be extracted is *M*_1_ = *N*−*N*_*g*2_ for group-1, and *M*_2_ = *N*−*N*_*g*1_ for group-2. Consequently, *N, N*_*g*1_, *and N*_*g*2_ should be set such that *N* − *N*_*g*2_ ≥ *K*_1_ and *N* − *N*_*g*1_ ≥ *K*_2_.

Equation (3) gives the independent component factorization for the aggregate matrix, with the additional constraint that two blocks of the new mixing matrix should be zero. This constraint can be integrated into the FastICA cost function (Hyvarinen, 1999) using the Lagrange multipliers method (Lang, 1987), and then be optimized using Newton's method (for further details on solving these equations, see Vahdat et al., 2012). This resolution provides the SSICA iterative formula (see also Equation 2.19 in Vahdat et al., 2012), which allows, simultaneously, maximization of independence at the component level and orthogonality of the specific components at the group level.

### Labeling of the specific components based on the back-reconstruction

In the current paper, to ensure that the iterative algorithm of SSICA starts from an initial point closer to the local maxima and consequently converges faster when applied to high dimensional fMRI data, we modified the procedure for labeling of the specific components at the very first iteration of the algorithm. Originally, at each SSICA iteration, the mixing matrix was evaluated to determine those components that have zero (or relatively small) elements in one group of subjects (refer to Equation 2.23 in Vahdat et al., 2012). Then these components were treated as potential specific components and would enter the SSICA iterative algorithm.

Here, we propose that, at the very first iteration of the algorithm, the component membership (shared, specific group-1, or specific group-2) be evaluated by comparing the estimated component's power across the two groups of subjects. To do so, we use the percent variance accounted for (PVAF) as an estimate of the percentage of variability that a single component (*c*^*th*^) can explain in an individual's data matrix, as follows:

(4) $$\begin{array}{l} {\left(PVAF_{i}^{j}\right)}_{c}=\left\{\left(1-\frac{var\left(Y_{i}^{j}-{\left({Ŷ}_{i}^{j}\right)}_{c}\right)}{var\left(Y_{i}^{j}\right)}\right)\times 100\right\}\% \end{array}$$

Where ${\left({Ŷ}_{i}^{j}\right)}_{c}$ is the projection of the *c*^*th*^ component on the data of subject *i* in group *j* calculated based on the inverse of Equation (B.9) (see Appendix B in Supplementary Material): ${\left({Ŷ}_{i}^{j}\right)}_{c}={\left(F_{i}^{j}\right)}^{+}B_{i}^{j}D^{j}{Â}_{c}Ŝ$, where Â_*c*_ is constructed by making all, except for the *c*^*th*^, columns of mixing matrix zero, and (.)^+^ denotes the pseudo-inverse operator. *var*(.) denotes the average of variances of different rows of a matrix (average in time). It should be noted that in the subsequent iterations, however, the original faster algorithm for shared/specific component classification (as explained in Vahdat et al., 2012) was used.

So, at the first iteration of SSICA, for every component its subject-specific PVAF is calculated based on Equation (4). Then *t*-statistics are considered to compare the PVAF values between the two groups of subjects. Those components that have significantly greater PVAF values in one group of subjects will be labeled as specific for that group. The significance level was set to α = 0.05/*N* accounting for multiple comparisons using Bonferroni correction. As explained earlier, in SSICA the maximum number of specific components that can be extracted is set by the user (*N* − *N*_*g*2_ and *N* − *N*_*g*1_ components for group-1 and group-2, respectively). If the number of specific components in group-1 (or group-2) that passes α exceeds these maximum values, only the first *N* − *N*_*g*2_ (or *N* − *N*_*g*1_) components showing the most significant group differences (lowest *p*-values) will be labeled as specific.

### fMRI acquisition

To test the validity of SSICA on real fMRI datasets, two datasets were examined: (I) a hybrid fMRI dataset composed of patches added to real resting-state data (II) a dataset originating from a finger-tapping experiment with visual or auditory cues. Twelve right-handed healthy adults aged 18–24 were scanned at the Montreal Neurological Institute (MNI). The study was approved by the MNI ethics committee and subjects participated in the research after giving written informed consent. Functional images were continuously acquired using a 3T MR scanner (Siemens Trio, Germany) with a 32-channel head coil. MP-RAGE anatomical images were first acquired (1 mm slice thickness, 256 × 256 matrix; TE = 2.98 ms and TR = 2300 ms; flip angle 9°) and used for superposition of the functional images and inter-subject group co-registration. The functional data were acquired using a T2^*^-weighted EPI sequence (3.5 × 3.5 × 3.5 mm voxels, 39 slices, 64 × 64 matrix; TE = 25 ms and TR = 2000 ms; flip angle 70°). Each subject's functional scan comprised of three conditions: visual cued finger-tapping (VFT), auditory cued finger-tapping (AFT), and resting-state (RS). Each condition was repeated over 2 runs of 200 s each. Resting-state was defined as a state of relaxed wakefulness when subjects had their eyes open and were instructed to focus on a cross in the middle of a white screen. In the VFT condition, subjects were required to perform finger tapping at a constant pace in accordance with visual cues. Subjects were instructed to tap their right thumb to index or middle finger in accordance with a color-coded blinking (200 ms on period) circle presented at 1 Hz frequency in a pseudo-random sequence with the same probability of occurrence for each finger. For the AFT task, subjects tapped their right thumb to index or middle finger as explained above, but in accordance with an auditory tone having a frequency switching between two values. Using a computer playback system, auditory tones (duration of 200 ms) were presented binaurally through a headset every 1 s. In all conditions subjects were asked to focus on a cross in the middle of a white screen.

### fMRI preprocessing

Data processing was carried out using the FMRIB Software Library (FSL), www.fmrib.ox.ac.uk, Oxford U.K., FSL version 4.1 (Beckmann et al., 2003). The following preprocessing steps were applied to the functional data: (1) removal of the first two volumes of each run to allow for equilibrium magnetization, (2) slice timing correction using Fourier-space time-series phase-shifting, (3) non-brain tissue removal, (4) rigid-body motion correction, (5) global intensity normalization of all volumes of each run as implemented in FSL, (6) spatial smoothing using a Gaussian kernel with 6 mm full width at half maximum, and (7) high-pass temporal filtering with cut off frequency of 0.01 Hz. Conversion of the low-resolution functional data to the average standard space (MNI152) involved two transformations. First, from low resolution EPI image to high-resolution T1-weighted structural image (using a 7 degree-of-freedom affine transformation), and second, from T1-weighted structural image to the average standard space (using a 12 degree-of-freedom linear affine transformation, voxel size = 2 × 2 × 2 mm). The preprocessed and MNI-transformed functional data were further sub-sampled into 4 mm isotropic space.

### Hybrid fMRI data generation

**Figure 2:**
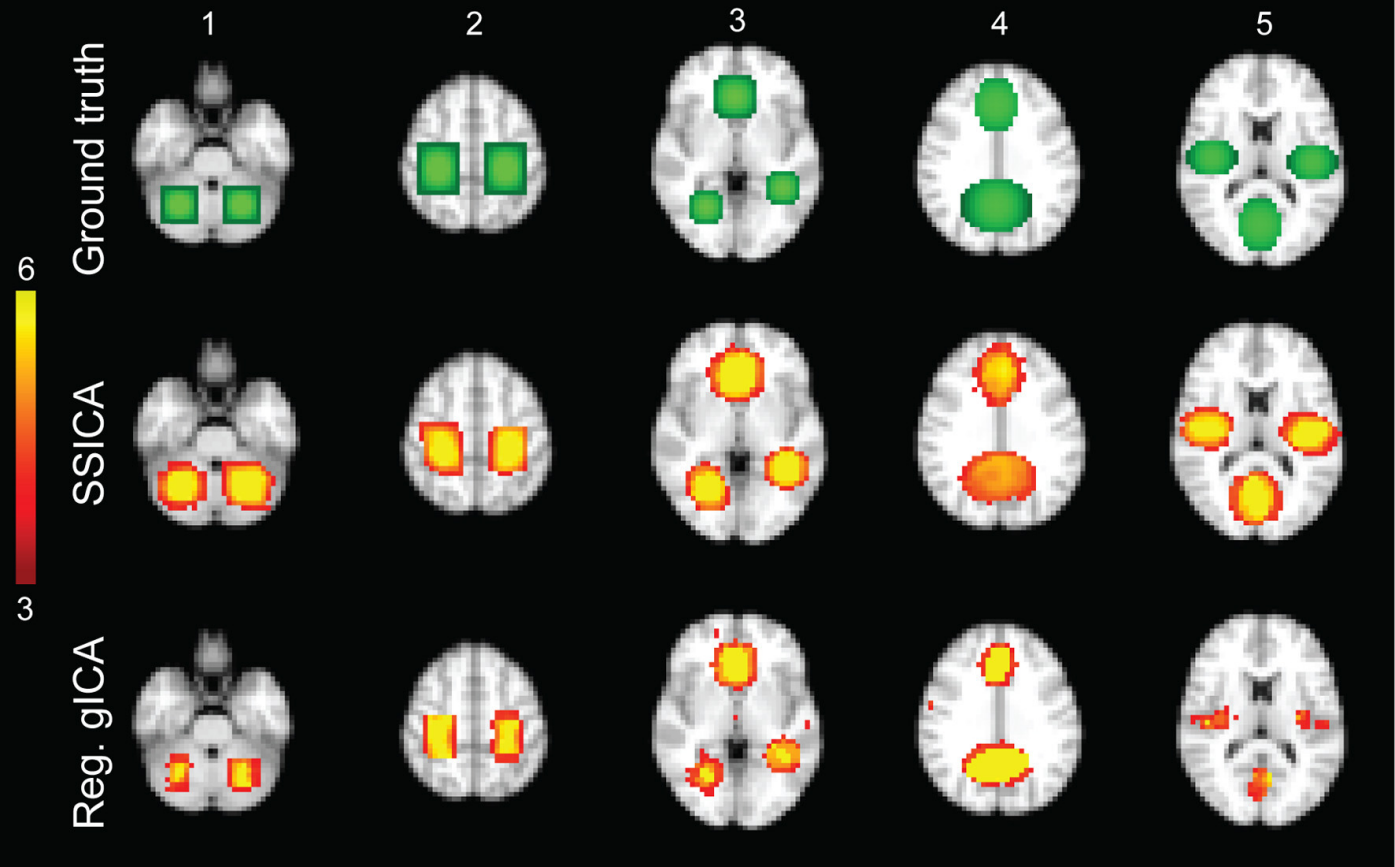
**Hybrid fMRI data generation**. Top row: the source patches used to generate the hybrid fMRI data set. From left to right, it illustrates the patches (or components), which are located in the cerebellum, sensorimotor area, anterior cingulate and lingual regions, paracingulate gyrus and precuneus, and bilateral opercular cortex and middle visual area. Middle and bottom rows show the *t*-statistics maps of those components, respectively, extracted by the SSICA and the regular time-concatenation gICA approach, which show the highest correlation with the ground truth patches. In this example, the 2 patches shown on columns 1 and 2 were specific to group-1, the 2 patches on columns 4 and 5 were specific to group-2 and the one on column 3 was shared between groups. The specific maps are generated using two-sample *t*-statistics, while the shared map is generated using one-sample *t*-statistics. The SSICA results are more similar to the ground truth specific patches compared to the regular gICA approach.

We randomly divided the twelve subjects into two groups, resulting in 12 resting-state functional runs (two runs for each of six subjects) in each group. To generate hybrid fMRI data, we added focal patches of activations to the sub-sampled normalized functional data of each subject during the resting-state condition. As shown in Figure 2 top row, five different patches configurations were generated. Each patch was actually composed of 2 or 3 distinct volumes of interest, consisting in clusters of neighboring voxels, which will be further denoted as blobs in this study. So each patch was composed of 2 or 3 blobs.

The spatial intensity distribution within each blob was set using a gradient of relative amplitudes ranging from 0.7 to 1.3, increasing from the outer to the central region, with averaged relative amplitude of one. We refer to the spatial intensity distribution of each patch as the ground-truth *s*_*p*_ (a spatial map of M voxels). This spatial intensity distribution is multiplied by a time course and then added to the real resting-state fMRI data of each subject. For each subject and each patch, the simulated time course was defined separately using different realizations of standard zero-mean Gaussian signals band-pass filtered between 0.01 and 0.1 Hz to mimic the neuronal-related portion of resting-state BOLD fMRI time series (Fox and Raichle, 2007). The variance of the Gaussian distribution used to generate patch time-series in each subject was set to the average variance of all the brain voxels' time series during resting state. The power of each patch was defined as the ratio of that patch's time-series variance to the average variance of all the brain voxels' time series during resting state. To generate simulations at different SNRs, we multiplied the simulated patch's time-series by a factor, called the signal to noise ratio (*SNR*). In the simulations, *SNR* was varied from 0.5 to 1 (resulting in simulated patches' power of 0.5–1, respectively) in steps of 0.1 to have a realistic model of the added activities. At a *SNR* of 1, the patches were always found within the 20 strongest (measured by the amount of explained variance) ICA components. As a comparison, the average power values calculated from the extracted time-series of the default mode, auditory and visual networks during the resting-state conditions were 1.04, 1.01, and 1.12, respectively. Note that for generating a specific patch of one group, the *SNR* of that patch was set to zero in the subjects of the opposite group, while for the shared patches the *SNR* was non-zero for all subjects. In our simulations, we randomly generated many different realizations by setting each patch as either specific or shared. To account for anatomical variability between subjects, we introduced axial translations in the patch location. We shifted the embedded patches of each subject by a value randomly selected from –*n*/2 to *n*/2 voxels in the axial plane (voxel size = 4 mm isotropic; *anatomical noise* up to *n* = 6 voxels, i.e., 12 mm in each direction).

### Patch extraction performance

To investigate reconstruction performance of the SSICA, we measured reconstruction error and similarity between the ground-truth signals (the simulated patches: *s*_*p*_) and the extracted patches (${\tilde{s}}_{p}$) by calculating the spatial root mean square error (RMSE) and the spatial correlation *r*^2^ between *s*_*p*_ and ${\tilde{s}}_{p}$, defined as:

(5) $$RMSE=\sqrt{\frac{1}{M}\sum_{m=1}^{M}{\left(s_{pm}-{\tilde{s}}_{pm}\right)}^{2}}s,r^{2}={\left[corr\left(s_{p},{\tilde{s}}_{p}\right)\right]}^{2}$$

Where, *s*_*pm*_ and ${\tilde{s}}_{pm}$ correspond to the *m*th voxel of the *p*th ground-truth patch and its corresponding extracted component, respectively. *M* is the number of voxels, and corr(.) represents Pearson correlation. Note that, the *Z*-score spatial maps (spatially demeaned and divided by standard deviation of M voxels within a brain mask) of the ground-truth and the extracted components were used to calculate the RMSE and *r*^2^ measures. Also, in order to acquire a clean map of activation and to be more sensitive to areas of significant activation, the *Z*-score spatial maps were first thresholded at |*Z*| > 2.3 (*p* < 0.01, uncorrected).

To quantify performance of shared and specific components classification, receiver operating characteristics (ROC) analysis was considered. We had to adapt ROC methodology for our evaluation and comparison between SSICA and gICA. To do so, we first defined as true positive class labels the simulated shared components and as true negative class labels the simulated specific components of the ground truth. In this analysis, we also arbitrarily selected the templates of three highly-reported resting-state networks including default mode, auditory, and visual networks, based on a previous study of resting-state ICA analysis (Damoiseaux et al., 2006), and included them as ground-truth maps of true positive class labels since we expect them to be present in our acquired resting-state data as well.

Therefore, for the five patches and the three resting-state templates, we identified those extracted components, which had the highest spatial correlation *r*^2^ with their corresponding ground-truth templates. For each of those components identified by SSICA, it was labeled as predicted positive when classified as either a shared component or as one of the three resting state networks. Consequently, it was labeled as predicted negative when classified as a specific component for one group.

For SSICA, there is actually an explicit threshold used to classify every component as shared or specific and this threshold is applied at every iteration of the SSICA algorithm (see Equation 2.23 in Vahdat et al., 2012 for more details). Therefore, we ran SSICA multiple times with 20 different *threshold* values ranging from 0 to 1, to get the classification performance at different points along the ROC curve.

For the regular gICA approach, the “predicted class label” was defined using the following procedure. First, the back-reconstruction method implemented in GIFT software was used on the group-level maps generated by applying ICA on the time-concatenated data of all subjects, in order to assess the contribution of every subject to every component detected using gICA (see Appendix A in Supplementary Material for further details). Afterwards, for each identified component, two-sample *t*-statistics were performed on back-reconstructed data to contrast the subject-specific maps of the two groups (significant voxels were defined for a *p*-value < α; uncorrected, α = 0.01 or 0.001). Two different values for α were considered to ensure that the pattern of results was not dependent on the choice of α. The predicted class label for each identified component was set positive, i.e., more likely corresponding to a shared component, if the number of significantly active voxels in the two-sample *t*-test map was less than a threshold, thus suggesting no group differences. On the other hand, the predicted class label was set negative, i.e., more likely corresponding to a specific component, when the number of significant voxels (positive or negative *t*-values) was larger than a threshold, thus suggesting a significant group difference. Consequently, when building the whole ROC curve, classification performance was assessed by varying this threshold (the minimum number of active voxels in the two-sample *t*-test map which reveals a non-zero difference map) over its possible range (ranging from zero to maximum number of activated voxels across all components and subjects).

The optimal point in the ROC curve (a point with the least sum of squared false positive and false negative ratios) was identified to measure the best classifier performance in the SSICA and the regular gICA method.

### Robustness of SSICA to the number of extracted specific components

As described in Section Standard ICA and Group-Level ICA, the maximum number of specific components that can be extracted in each group depends on the second and third data reduction steps (maximum *M*_1_ = *N* − *N*_*g*2_ specific components for group-1, and *M*_2_ = *N* − *N*_*g*1_ for group 2). To investigate the robustness of SSICA to various choices of these parameters, we assessed the performance when *M*_1_ (or *M*_2_) was set less or more than the actual number of simulated specific components, *K*_*g*1_ (or *K*_*g*2_).

For the condition in which *M*_1_ < *K*_*g*1_ (or *M*_2_ < *K*_*g*2_), we generated several datasets by randomly assigning two patches as specific component in each group (*K*_*g*1_ = *K*_*g*2_ = 2) and one patch as shared component, but asked the algorithm to extract, at most, one specific component per group (*M*_1_ = *M*_2_ = 1). We examined whether the SSICA shows some preference in extracting one specific component over the other one, according to their strengths. To evaluate this, the strength, defined as the patch *SNR*, of one of the two specific components in each group was set *m* times larger than the strength of the other one. We will refer to *m* simulation parameter as the “*unbalance ratio*.” This simulation was designed to examine whether SSICA classifies the strongest among the two patches as specific, randomly selects one based on initialization, or pools the two together in one component.

To assess such behavior of the algorithm, we defined a *similarity ratio* to quantify the degree to which the extracted specific component correlated to the weak or to the strong simulated patch as follows:

(6) $$\begin{array}{l} similarity\;ratio=\frac{r_{1}}{\sqrt{{r_{1}}^{2}+{r_{2}}^{2}}} \end{array}$$

Where *r*_1_ (respectively, *r*_2_) represents the spatial correlation between the extracted specific component and the strong (respectively, the weak) simulated patch.

For the condition in which *M*_1_ > *K*_*g*1_ (or *M*_2_ > *K*_*g*2_), we generated several datasets by randomly assigning one patch as specific component for each group *K*_*g*1_ = *K*_*g*2_ = 1 and the rest as shared components, but asked the algorithm to extract up to two specific components per group (*M*_1_ = *M*_2_ = 2). In this situation, it is possible that SSICA split the specific patch into 2 specific components, depending on the degree of temporal similarity or correlation among blobs within the simulated patch. To quantify this effect, we introduced some variability among the different blobs of the same patch by adding different realizations of zero-mean uncorrelated Gaussian noise to the time course of each blob. Accordingly, “within-component variability,” *k*, was defined as the ratio of Gaussian noise variance to the average variance of all the brain voxels' time series during resting state. In such a condition, we might observe three types of results: (i) *repetition*: the specific patch is repeated in both components extracted as specific, (ii) *split*: the specific patch is splitted between the extracted specific components or (iii) *noise*: the specific patch is represented in just one of the extracted specific components (hence the other one is an unrelated noise component). To distinguish these conditions, we propose the following metric, named *splitting factor*, which we estimated for each group:

(7) $$\begin{array}{r} splitting\;factor=\max_{l\in blobs}\left(\frac{mean\left(Z_{l,weak}\right)}{mean\left(Z_{l,weak}\right)+mean\left(Z_{l,strong}\right)}\right) \end{array}$$

Among the two extracted specific components in each group, *weak* (respectively, *strong*) refers to the one which shows a lower (respectively, higher) correlation with the ground-truth specific patch. *mean*(*Z*_*l, weak*_) denotes the averaged *Z*-score values within the *l*-th blob of the weak component, and *mean*(*Z*_*l, strong*_) denotes the averaged *Z*-score values within the *l*-th blob of the strong component. This ratio ranges between 0 and 1. For the values around 0.5, the weak component includes at least one blob that is already presented in the strong one [cf. the repetition condition (i)]. Values close to one represent the situation in which there is at least one blob in the weak component that is not present in the strong one [cf. the split condition (ii)]. Finally, values close to zero indicate that no blob of the specific patch was presented in the weak component [cf. noise condition (iii)].

### Robustness of SSICA with Regards to the Orthogonally Assumptions

In fMRI connectivity analysis, two different experimental conditions may activate the same set of brain clusters, but with different relative weighting of voxels within each activation cluster. This represents a case in which the specific networks of each condition (group) have overlapping activated clusters across conditions (group); hence, the projection of some specific networks in the opposite group might be non-zero. Furthermore, in fMRI datasets a brain network that represents the pattern of differences across groups is not usually fully absent in one of the groups, but rather less intense. Nevertheless, the fact that the Lagrange multiplier approach considered for the group orthogonality constraint in SSICA aims at minimizing the projection magnitude of the specific components does not mean that these specific components should have zero magnitude in the “opposite” group. But, practically, it ensures that, when the convergence is achieved, the magnitude of specific components is significantly lower in the “opposite” than in the “matching” group.

To test this idea, we generated different datasets in which some patches were embedded in both groups but with different strengths. We manipulated the strength of these “partially-specific” patches by setting to 1 the *SNR* value in the matching group, and from 0.1 to 0.9 the SNR of the same component in the opposite group (named *power ratio*). We examined whether SSICA extracts and labels these partially-specific patches as shared or specific components when varying the *power ratio*.

### Analysis of finger-tapping fMRI dataset

First, we analyzed the fMRI data obtained during finger-tapping conditions cued by visual and auditory stimuli using a block-design general linear model (GLM) approach as implemented in the Feat software, part of FSL (see http://fsl.fmrib.ox.ac.uk/fsl/fslwiki/FEAT). Following the regular preprocessing steps explained in Section fMRI Preprocessing, VFT and AFT conditions were concatenated with the subsequent baseline RS conditions. Each run of AFT or VFT was considered as one task block and was contrasted with the RS runs in a GLM. For each subject, a fixed-effect analysis was performed to average the two runs of the same condition. Then, the subject-level statistics images were input to a group-level GLM to calculate the following contrasts: (VFT − RS) − (AFT − RS), (AFT − RS) − (VFT − RS), and (VFT − RS) + (AFT − RS). To find peaks of activity, a group level mixed-effects model corrected for multiple comparisons at the cluster level was estimated using the FEAT software (Z > 2.3, corrected using Gaussian random field theory, corrected cluster *p* < 0.05).

For ICA network analysis, all the runs of VFT and AFT conditions (24 runs per condition, 12 subjects) were time-concatenated. GIFT and SSICA were applied on these time-concatenated data. For a fair comparison, similar to what was used for the analysis of simulated patches, the FastICA algorithm (Hyvarinen, 1999) was selected as the ICA method used in GIFT software. The dimension of each individual subject's data was first reduced from 2 × 100 to 50 in both algorithms ($F_{i}^{j}$ projector). We selected this value as it accounted for at least 98% of variance (mean ± std = 98.6 ± 0.3) in every subject's data in our task-based dataset. At the group level, *N* = 30 components were extracted in both algorithms (*G* projector). In order to test the stability of our results with respect to the total number of extracted components, we did additional analyses by extracting 40 and 50 components at the group level in both SSICA and GIFT. According to our hypothesis based on the experimental design, we should only extract one specific component per condition. However, in order to be more flexible to the extraction of specific components, we set the number of specific components per condition (VFT and AFT) to 3. This implies setting the second level data reduction dimension to *N*_*g*1_ = *N*_*g*2_ = *N*−3 = 27 on the concatenated data of either VFT or AFT (*H*^*j*^ projector). Therefore, the size of each condition's concatenated data was first reduced from (12 × 50) to 27, and then the size of the concatenated conditions data was reduced from 2^*^27 to 30 at the third level PCA. The back reconstruction method followed by two-sample t-statistics was performed on the results of both algorithms to identify the specific networks corresponding to each condition (thresholded at *t* = 3.17, *p* < 0.005, uncorrected).

In GIFT software, two-sample *t*-statistics were performed over a mask defined by the thresholded map (*t* > 1.5) of one-sample *t*-statistics for all subjects and conditions, as suggested by (Assaf et al., 2010), to explore results within the general activation map of the selected component only.

### Analysis of the consistency of the extracted specific components in task fMRI

The SSICA requires as input the number of networks specific to each group but the true value of this number is not known a priori, especially when dealing with real data. Following the methodology already proposed in our previous study (Maneshi et al., 2014), to test the consistency of the extracted specific components in task fMRI dataset, we decided to apply SSICA multiple times with different initializations and numbers of extracted specific components. Considering four possible values for the number of specific components (while excluding the situation where 0 specific networks are extracted in both conditions) and repeating the whole procedure five times (to account for the effect of the ICA initialization in algorithmic instability Himberg et al., 2004), we ended up with 15 × 5 = 75 SSICA estimations for further analysis.

The clustering method proposed by Hyvarinen and Ramkumar (2013) was further used to find the most reliable specific networks among all the identified specific components in each condition. The outputs of this analysis were several clusters of specific components with the highest within-cluster similarities and the lowest between-clusters similarities (see Maneshi et al., 2014 for further explanation regarding the basis of this clustering method). To obtain a representation of each cluster, all the components within that cluster were averaged. Results, overlaid on standard MNI152 at 1 mm resolution for visualization purposes, were then thresholded (*Z* > 2.3) and labeled according to the Harvard–Oxford cortical and subcortical (Desikan et al., 2006), and Juelich histological atlases (Eickhoff et al., 2005).

### Analysis of the specificity of the extracted specific networks in task fMRI

In order to quantify the degree of specificity of each specific network (that is, the ratio of explained power in the matching group in contrast to the opposite group), we calculated, for each individual, a measure of functional connectivity in each detected reliable specific network (visual or auditory) and compared it across conditions. Here, we defined the strength of functional connectivity within a network based on the power of its corresponding time-course in the frequency band of the signal [0–0.25 Hz; the highest identifiable frequency is 0.5 × TR (Nyquist frequency)]. For each specific network and each subject, we used the subject's fMRI data (dependent variable) and the network's spatial map (independent variable) in a GLM to find one associated time-course per network and subject. We then used power spectrum analysis (with the standard Hamming window as implemented in MATLAB) to assess the power of this estimated time-course within the 0–0.25 Hz frequency band. For each condition and each specific network, power was averaged across subjects within that condition to calculate the strength of functional connectivity within that network.

## Results

### Hybrid fMRI data analysis

In simulations using hybrid fMRI data, different combinations of patches as shared or specific components and with various values of *anatomical noise* and *SNR* were generated (see Section Hybrid fMRI Data Generation for Details). In all analyses, the FastICA algorithm was applied on the sphered data using the hyperbolic tangent as the derivative of contrast function and the symmetric algorithm as the decorrelation approach (Hyvarinen, 1999). Figure 2 illustrates the results of the SSICA and the regular gICA approaches on a sample SSICA run using *anatomical noise* = 2 voxels and *SNR* = 1. Figure 2 top row shows the ground-truth patches as described before. In this example, from the left, the first two patches were embedded as specific components of group-1, the middle patch as shared component between groups, and the last two as specific components of group-2. Figure 2 middle and bottom rows show the components extracted by applying respectively the SSICA and the regular gICA approaches (using time-concatenation and back-reconstruction methods; see Section Labeling of the Specific Components Based on the Back-Reconstruction). For each method, components exhibited the highest spatial correlation *r*^2^ with the ground truth patches are presented. As illustrated in this example, the spatial maps of the specific patches are reconstructed more accurately in the SSICA compared to the regular gICA approach. Also, unlike SSICA, the gICA approach resulted in underestimation of the spatial extent of some blobs together with some false positive activated clusters outside the patch boundaries. For each method, we also identified and compared several highly-reproducible resting-state networks (including default mode network, visual, motor, and auditory) to examine the similarity of SSICA and the regular gICA in extracting other shared networks. The results of this analysis, shown in Supplementary Figure 1, suggest that both methods produced highly comparable connectivity maps related to common resting-state networks.

#### Quantitative evaluation of patch extraction performance

**Figure 3:**
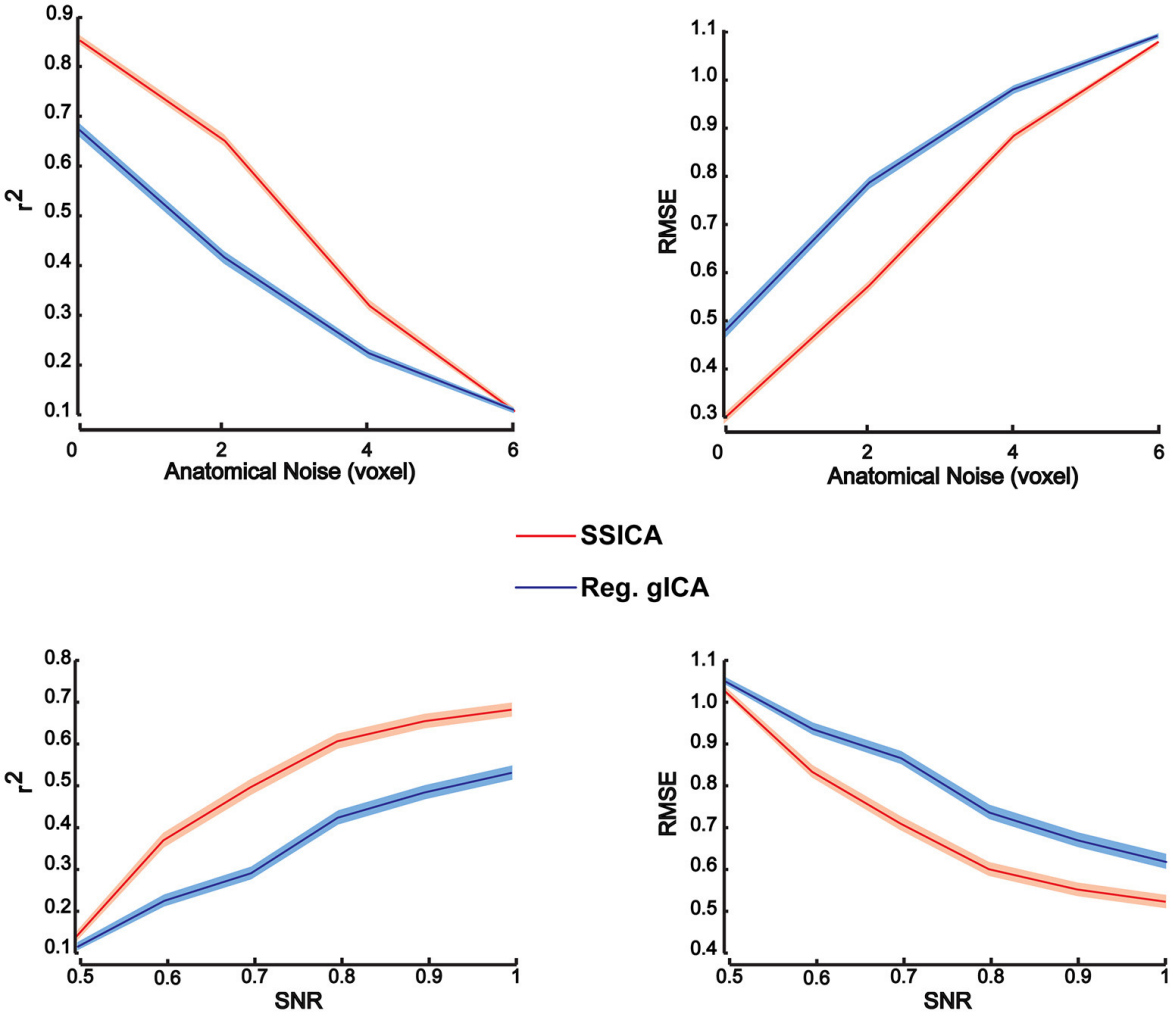
**Patch extraction performance**. Figure shows *r*^2^ (left panels) and RMSE (right panels) averaged over all extracted patches and permutations at different *SNR* and *anatomical noise* levels. At each *SNR* and *anatomical noise* level, the curves show the mean performance averaged over 2000 (100 permutations × 4 anatomical noise level × 5 patches) and 3000 (100 permutations × 6 *SNR* levels × 5 patches) extracted patches, respectively. The SSICA outperforms the regular approach, especially in cases where the power of the specific component is low or where the between subject variability in location of a component is high. Shaded area indicates ± standard error of the mean (sem).

To investigate patch reconstruction performance, RMSE and *r*^2^ measures were calculated and averaged over the results of the SSICA and the regular gICA approach on a large number of hybrid fMRI data sets. Data sets were generated using various combinations of patches as shared and specific components (100 random permutations of up to 2 specific patches per group) at different *anatomical noise* levels (*n* = 0, 1, 2, 3 voxels) and with different *SNR* values (0.5–1 in 0.1 steps). In total, 30 components were extracted by both algorithms. In SSICA, up to 3 components were allowed to be extracted as specific component per group. Among all extracted components, the five showing the highest correlation with the source patches were selected to estimate extraction performance using the metric defined in Equation (5). Figure 3 illustrates RMSE and *r*^2^ averaged over all extracted patches and permutations at different *SNR* and *anatomical noise* levels, for the SSICA in red and the regular gICA approach in blue. At each *SNR* and *anatomical noise* level, the curves show the mean performance averaged over 2000 (100 permutations × 4 *anatomical noise* level × 5 patches) and 3000 (100 permutations × 6 *SNR* levels × 5 patches) extracted patches, respectively. The SSICA outperforms the regular gICA approach, especially in cases where the strength of the desired component was low, or where the between subject variability in the location of the desired component (as expected when registration errors are still present) was high.

#### Patch classification performance

**Figure 4:**
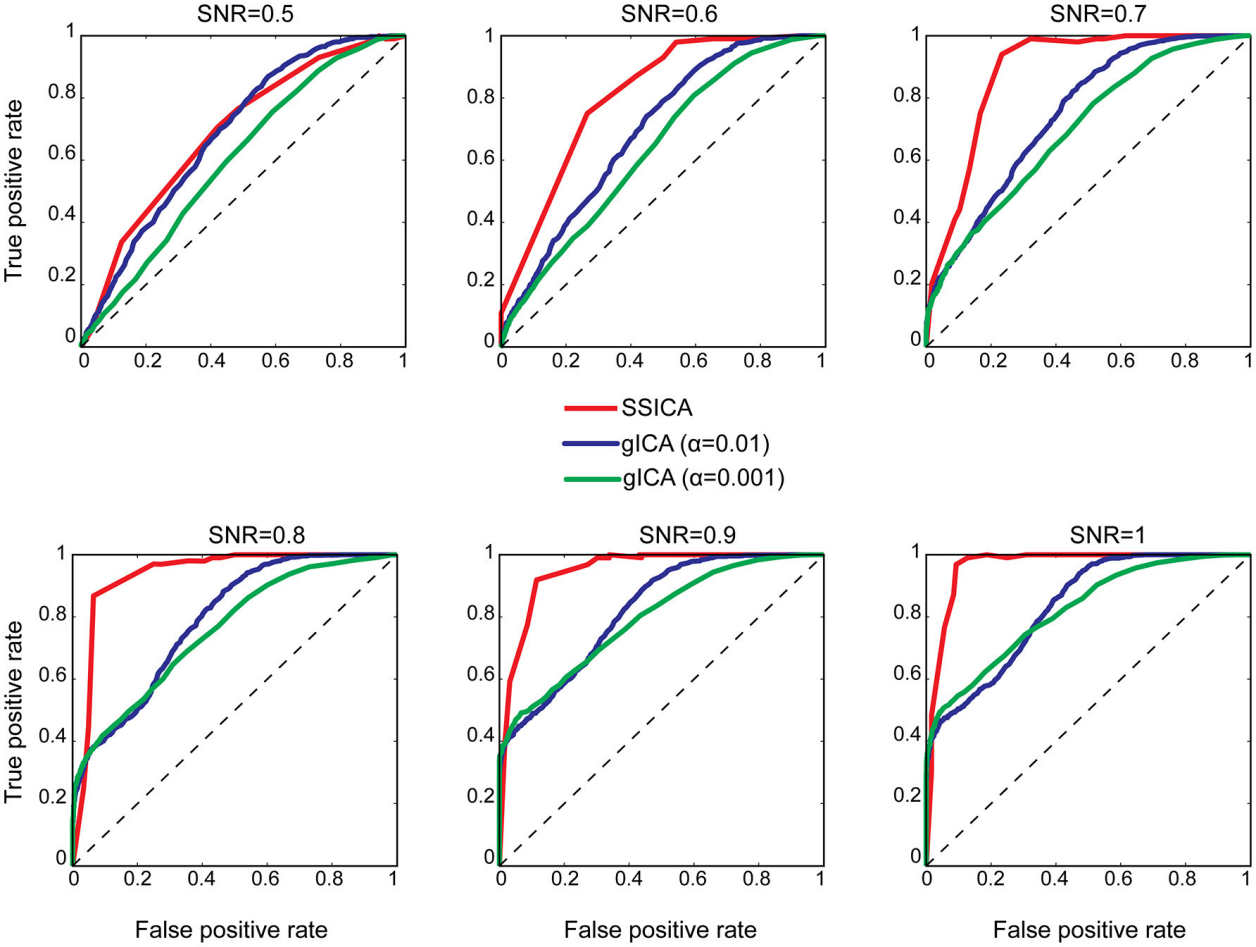
**ROC curves representing the classification performance of shared and specific patches and three resting-state networks at different ***SNR*** values**. For the SSICA, the ROC curve at each SNR level is generated using classification of 3200 extracted components (5 permutations × 4 *anatomical noise* level × 20 threshold values × 8 patches). For the regular approach, as there is no internal threshold, the ROC curve at each SNR level is generated using classification of 3200 extracted components (100 permutations × 4 *anatomical noise* level × 8 patches). RG01 and RG001 stand for the regular gICA approach with α = 0.01 and α = 0.001, respectively.

To investigate classification performance of shared and specific patches classification, ROC analysis was used as described in Section Patch Extraction Performance. The same above-mentioned parameters for *SNR* and *anatomical noise* levels, used in generating Figure 3, were used for the SSICA and the regular gICA approach. The ROC curves are based on the classification of the five simulated patches and three highly-reported resting-state networks (i.e., default mode, auditory, and visual networks) as described in Methods. Since in the SSICA the classification threshold needs to be specified in advance, we ran the algorithm multiple times (5 permutations) with 20 different threshold values ranging from 0 to 1, to estimate the ROC curve. Figure 4 shows the ROC curves at different *SNR* values for the SSICA in red, the regular gICA approach with α = 0.01 in blue, and the regular gICA approach with α = 0.001 in green (α is the two-sample *t*-test significance threshold). The ROC curves at each *SNR* level is generated using classification of 3200 (5 × 4 × 20 × 8) extracted components. Although the total number of active voxels in the difference map varies with the choice of α, as shown in Figure 4 the classification performance is quite stable using different α values, and is constantly superior in the SSICA compared to the regular approach. Particularly, the benefits of SSICA in the classification of specific patches (lower false positive rate) are greater at low *SNR* values.

#### SSICA with various numbers of extracted specific components

**Figure 5:**
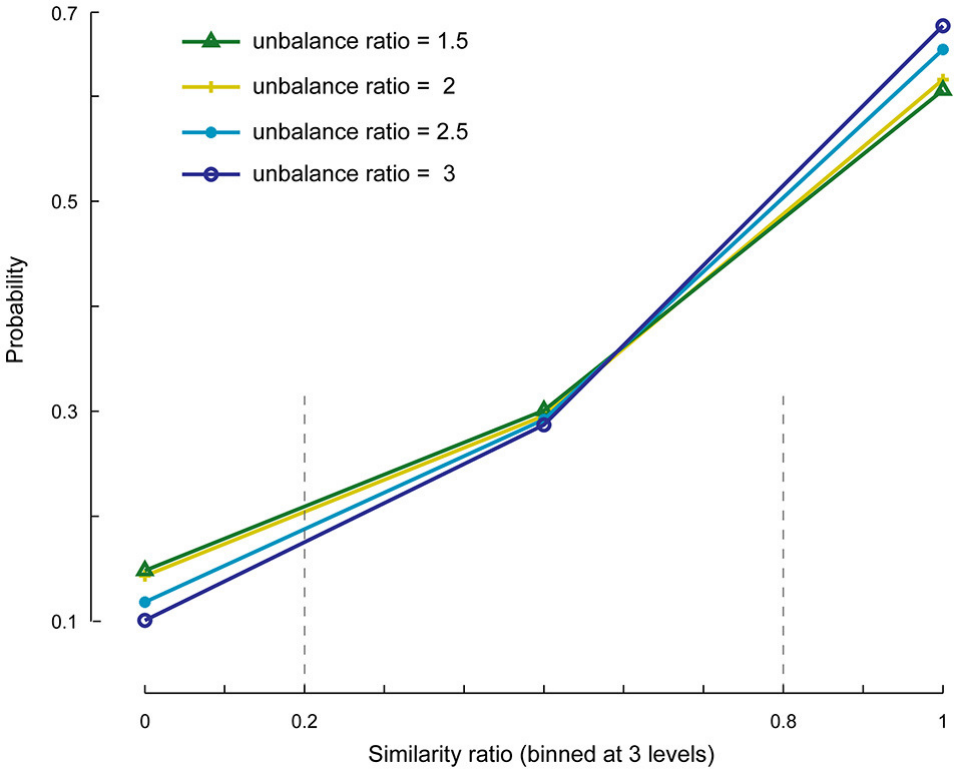
**Similarity ratio probability histograms at different unbalance ratio levels**. This figure shows the performance of SSICA when fewer specific components than the actual simulated components are extracted (*M*_1_ < *Kg*_1_ and *M*_2_ < *Kg*_2_). Similarity ratio is binned at three levels: low (0–0.2), middle (0.2–0.8), and high (0.8–1). Histograms are generated based a total of 480 extracted specific components (20 permutations × 4 *anatomical noise* levels × 6 *SNR* values) at each unbalance ratio level.

We investigated the cases in which the number of specific components is set to less or more than its actual value. First, we evaluated whether the SSICA extracts one or a combination of specific components when *M*_1_ < *K*_*g*1_ and *M*_2_ < *K*_*g*2_, and if this selection is influenced by the relative strengths of the components. To do so, we generated 1920 (20 × 4 × 6 × 4) hybrid fMRI datasets by various combinations of 2 specific patches embedded per group (20 random permutations), at different *anatomical noise* levels (*n* = 0, 1, 2, 3 voxels), with different *SNR* values (0.5–1 in 0.1 steps), and using various *unbalance ratios* (1.5–3 in steps of 0.5) as described in Section Robustness of SSICA to the Number of Extracted Specific Components. Whereas, two specific patches were simulated, up to one specific component per group was allowed to be extracted with SSICA. Figure 5 shows the *similarity ratio* probability histogram (Equation 6) calculated based on the results of SSICA at different unbalance ratios.

In order to make the interpretations easier, the similarity ratio was binned at three levels: low (0–0.2; corresponds to a case where the weaker simulated patch is extracted as the specific component), middle (0.2–0.8; where a mix of both patches is extracted as the specific component), and high (0.8–1; where the stronger patch is extracted as the specific component). The extraction of specific components is largely biased toward the stronger patch as represented by the values of *similarity ratio* close to one vs. zero (~60% as opposed to ~10% of the times). Also, ~30% of the times a combination of both specific components were extracted as a single specific component as represented by the values of *similarity ratio* between 0.2 and 0.8. As shown in Figure 5, by increasing the unbalance ratio the probability of extracting a weaker patch as specific component decreased, while the probability of extracting the stronger patch increased. This test indicates the preference of the SSICA algorithm to extract specific components based on their power, as opposed to a random initialization process. Although, one should note the possibility of merging two distinct specific components in a single specific component (due to inadequate number of allowed specific components) that may happen 30% of the times, regardless of the relative power of the two specific components.

**Figure 6:**
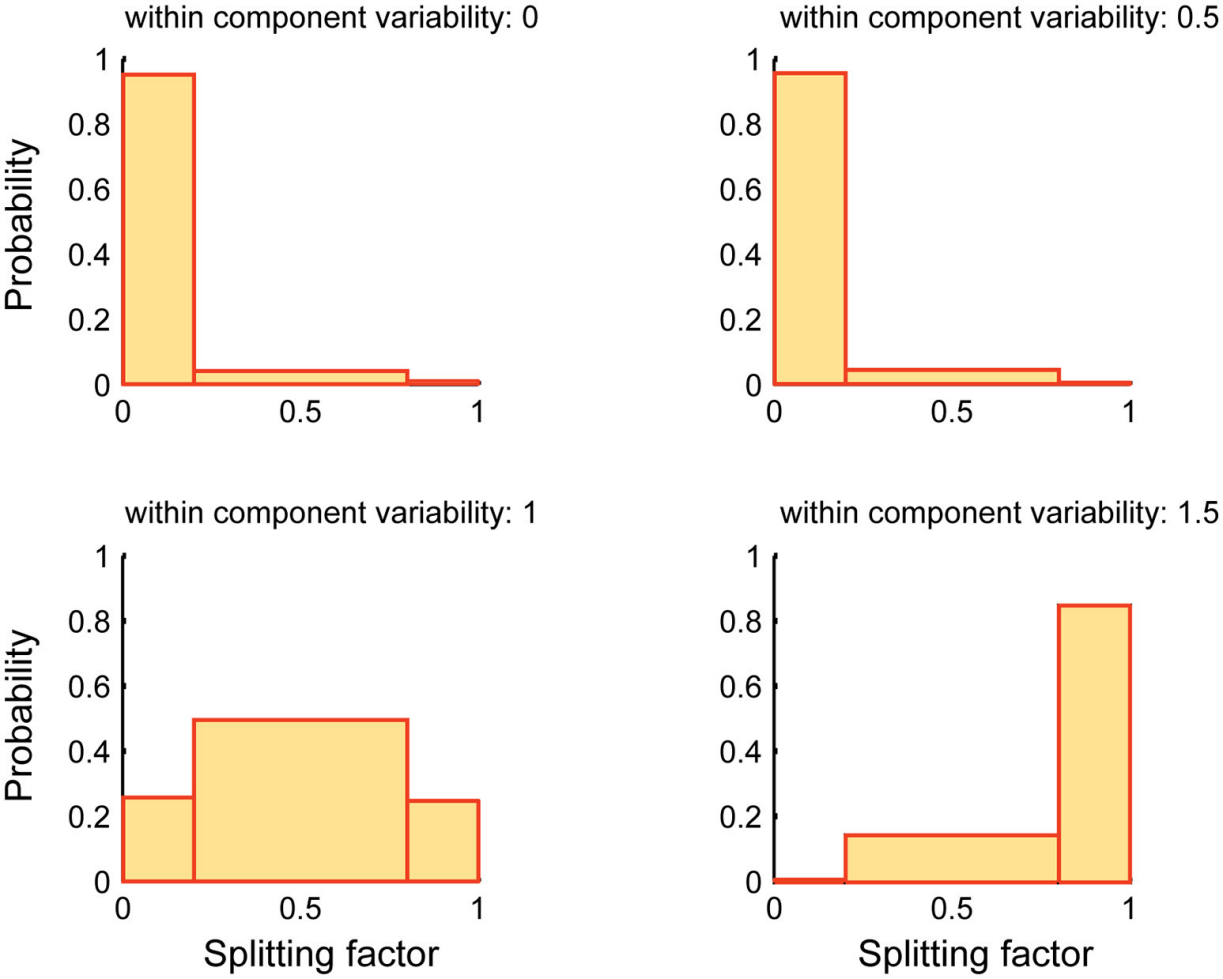
**Splitting factor probability histograms at different within-component variability levels**. This figure shows the performance of SSICA when more specific components than the actual simulated components are extracted (*M*_1_ > *Kg*_1_ and *M*_2_ > *Kg*_2_). Splitting factor is binned at three levels: low (0–0.2) (noise condition), middle (0.2–0.8; repetition condition), and high (0.8–1; split condition). Histograms are generated based a total of 480 pairs of specific components (20 permutations × 4 *anatomical noise* levels × 6 *SNR* values) at each within-component variability level.

Figure 6 reports the performance of SSICA when more specific components than embedded specific patches are extracted (*M*_1_ > *K*_*g*1_ and *M*_2_ > *K*_*g*2_). The *splitting factor* probability histograms are shown at different within-component variability levels (Equation 7). 1920 (20 × 4 × 6 × 4) hybrid fMRI datasets were generated by various combinations of one specific patch embedded per group and 3 shared patches (20 random permutations), at different *anatomical noise* levels (*n* = 0, 1, 2, 3 voxels), with different *SNR* values (0.5–1 in 0.1 steps), and using various within-component variability levels (*k* = 0–1.5 in 0.5 steps). Whereas, only one specific component per group was simulated in each configuration, two specific components per group were allowed to be extracted in the SSICA. Again, in order to make the interpretations easier, the splitting factor was binned at three levels: low (0–0.2; noise condition where the strong, but not the weak, extracted specific component is correlated with the simulated specific patch), middle (0.2–0.8; repetition condition where some blobs of the simulated specific patch is repeated in both the weak and strong extracted specific components), and high (0.8–1; split condition where the simulated specific patch is split into the two extracted specific components). As shown in Figure 6, at no or low within-component variability levels (*k* < 1), SSICA extracts all the blobs of the specific patch as one specific component, while the second component represents only extraction noise (cf. *splitting factor* around zero). At intermediate within-component variability levels (*k* = 1), SSICA still extracts one specific component which includes the whole patch's blobs, while the second component duplicates some of the blobs (cf. *splitting factor* around 0.5). At high within-component variability levels (*k* > 1), SSICA splits the specific patch into the two extracted specific components for each group (cf. *splitting factor* around one). This simulation demonstrates that depending on the degree of within component variability, SSICA extracts the specific patch exclusively, repeats it in several components, or split it across different components.

#### SSICA performance in partially-specific conditions

**Figure 7:**
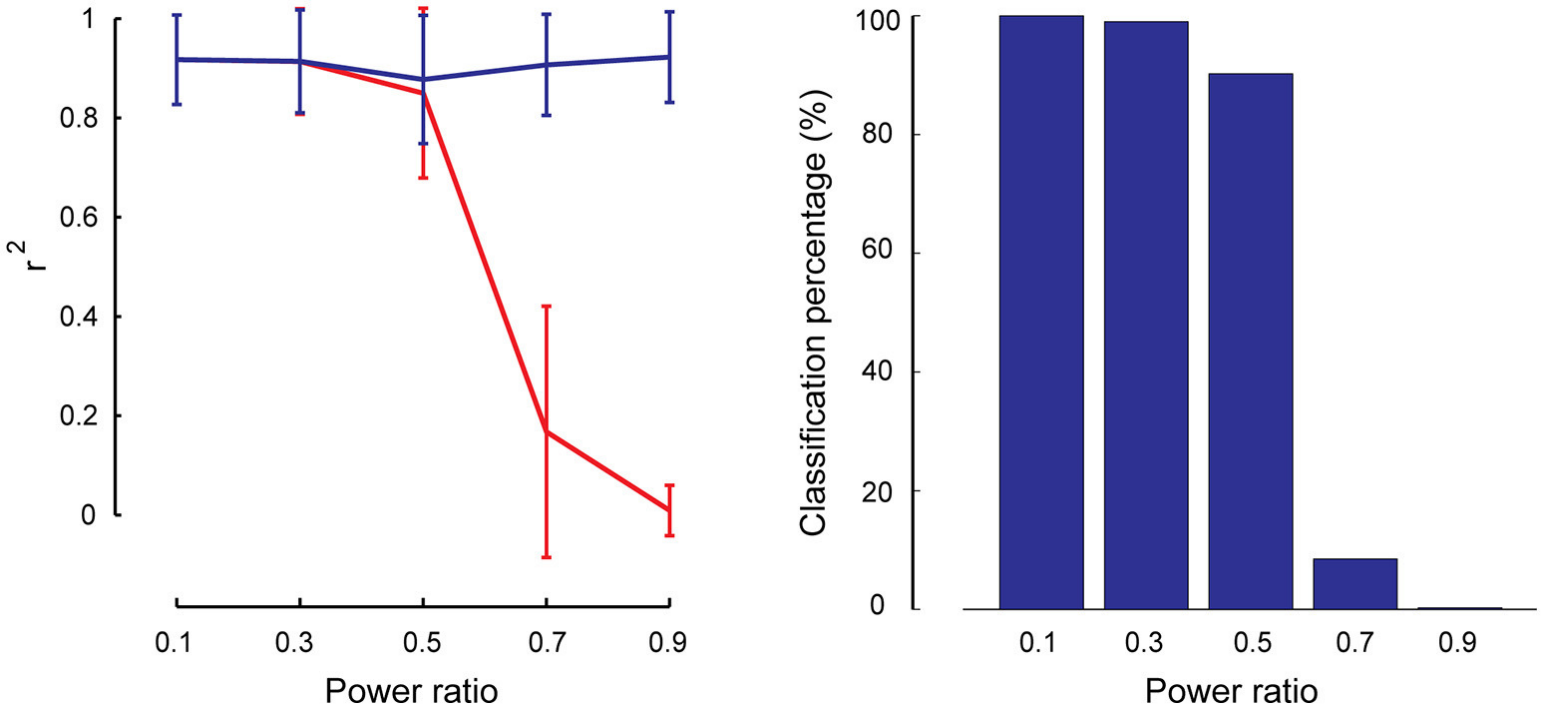
**Performance of SSICA in partially specific conditions**. Left panel shows the reconstruction performance of partially-specific patches as measured by *r*^2^ averaged over 600 extracted components (50 permutations × 4 *anatomical noise* levels × 3 partially-specific patches on average) at each power ratios. Power ratio is defined as the ratio of the simulated patch's SNR value in the opposite group to the matching group. Error bars indicate standard deviation. Blue and red curves show the average *r*^2^ when the matching component could be selected from all extracted components, and from just the specific components, respectively. Right panel shows the classification performance of the partially specific patches as the percentage of times in which the SSICA classified the partially- specific patches as a specific component, calculated at different power ratio levels.

As described in Section Robustness of SSICA with Regards to the Orthogonally Assumptions, partially-specific components were generated to simulate cases where a brain network is not totally missing within the opposite group (i.e., having rather a small but non-zero power), while it is significantly activated in the matching group. A total of 1000 (50 × 4 × 5) hybrid fMRI datasets were generated by various combinations of patches as shared and partially-specific components (50 random permutations of up to 2 partially-specific patches per group), at different *anatomical noise* levels (*n* = 0, 1, 2, 3 voxels), and using different *power ratios* which specify the relative *SNR* of the partially-specific components between groups (0.1–0.9 in 0.2 steps). We calculated the reconstruction performance of the partially-specific components at different *power ratios* by calculating *r*^2^ between the corresponding patch and the most similar extracted component either among all the components, or just among the specific ones. Figure 7 left panel shows these results based on the entire extracted components in blue and based on the specific components in red. As expected, SSICA is able to extract the partially-specific components with high accuracy either as shared or specific component (*r*^2^ ≈ 0.9 left panel, blue curve). However, only at power ratios ≤0.5, the corresponding extracted component was among the specific components. At power ratios more than 0.5, SSICA labeled the corresponding partially-specific component most often as a shared component. To better quantify the classification performance, the percentage of times in which the SSICA classified the partially-specific patches as specific was calculated (i.e., the number of cases in which the best correlated component with the patch was among the extracted specific components). Figure 7 right panel shows classification performance of the partially-specific components averaged over all hybrid simulated datasets at different relative SNR values. The classification performance curve closely mimics that of the reconstruction performance among the specific components. Overall, this simulation demonstrates that SSICA has the flexibility to extract and label partially specific components as specific when the component's *SNR* is at least twice larger in one group than in the other. In situations where a component's power is more or less comparable across groups (power ratio between 0.5 and 1), it may be more natural to label this component as shared and then to evaluate differences in the power of shared components across groups.

### Analysis of finger-tapping dataset

**Figure 8:**
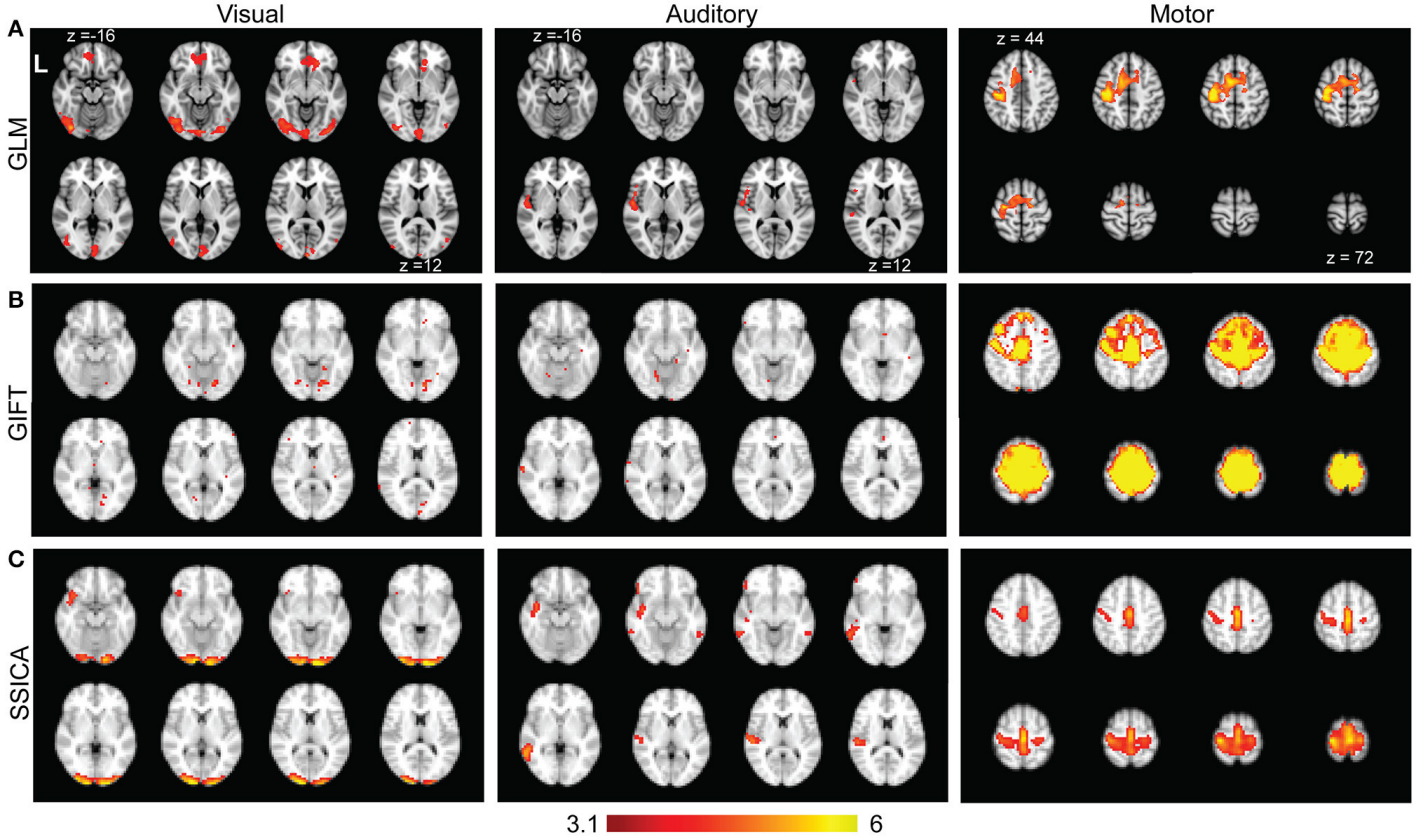
**Results of GLM, GIFT, and SSICA in extracting active clusters during finger-tapping task**. **(A)** *Z*-score maps showing active clusters for different contrasts during finger-tapping tasks using GLM analysis. Only the slices with significant activity are shown. The left (Visual), middle (Auditory), and right (Motor) panels show the significantly active clusters for (VFT − RS) − (AFT − RS), (AFT − RS) − (VFT − RS), and (AFT − RS) + (VFT − RS) contrasts, respectively (corrected using Gaussian random field theory, cluster level *p* < 0.05). **(B)** Left, middle, and right panels show the results of GIFT software for the contrasts of (VFT − AFT) on visual, (AFT − VFT) on auditory, and (AFT + VFT) on motor network, respectively. **(C)** Left, middle and right panels show t-statistics applied on back-reconstructed SSICA maps corresponding to specific components of VFT, AFT, and a shared component comprised of motor network, respectively. The statistical maps reported in **(A)** are generated based on fMRI data in AFT, VFT, and RS conditions, while the statistical maps in **(B)** and **(C)** are calculated based on the data in AFT and VFT conditions only. All ICA maps are back-reconstructed and resulting group level t-maps were thresholded at *t* = 3.17, *p* < 0.005, uncorrected.

Figure 8A shows the results of standard GLM analysis as described in Section Analysis of Finger-Tapping fMRI Dataset on the fMRI data acquired during finger-tapping conditions cued with visual and auditory stimuli. As expected, the between-condition contrasts of (VFT − RS) − (AFT − RS) and (AFT − RS) − (VFT − RS) included mostly clusters of activity in the visual and auditory areas of the brain, respectively. The auditory network was found unilateral within the right hemisphere. The contrast representing the average activity during both conditions compared to the baseline, i.e., (AFT − RS) + (VFT − RS), included clusters in the motor network comprising M1, SI, premotor cortex, and supplementary motor area.

Time concatenation gICA followed by back-reconstruction as implemented in GIFT software was applied to the AFT and VFT data, as described in Section Analysis of Finger-Tapping fMRI Dataset. Two sample *t*-statistics were applied on the results of back-reconstruction to obtain the map of differences between the two conditions for each extracted component. Among all the components those showing a significant difference map (either positive or negative) were selected. Figure 8B, left and middle panels show those with the highest spatial correlation with the visual and auditory networks templates, respectively. Beside some sparse activated voxels which were spread all over the brain, no component comprised of any significant cluster in either visual or auditory areas were detected, even when two-sample *t*-maps where thresholded with a more liberal threshold of *t* > 2.5. Therefore, although some parts of the auditory and visual networks were found as shared between groups, no network showing a significant contrast in AFT task vs. VFT (and vice versa) was found using the GIFT method. Figure 8B, right panel shows the results of one-sample *t*-statistics on a motor network. As shown, the standard gICA approach as implemented in GIFT software was able to extract shared networks between the two conditions (there might be some overestimation of the extent of the motor network using the GIFT method, compared with the motor network resulted from the GLM analysis).

Figure 8C shows the results of applying SSICA to the AFT and VFT conditions followed by the extended back-reconstruction method to obtain *t*-statistics maps corresponding to shared and specific components as described in Methods. Although up to 3 specific components per condition were allowed, the SSICA only resulted in one specific component for each condition. Figure 8C, left panel shows the specific component corresponding to VFT, which mainly includes primary and secondary visual areas. The middle panel shows the specific component corresponding to AFT, comprised of left superior and middle temporal areas including the Heschl gyrus. The right panel in Figure 8C shows one of the shared networks including bilateral motor and premotor areas. The SSICA results are consistent with the two cueing conditions of the finger-tapping task and with the results of GLM based analysis (Figure 8A). A similar overall analysis was also performed when selecting 40 (or 50) components at the group-level, and generally a very similar pattern of results was obtained using SSICA and GIFT (results not shown).

### Analysis of consistency and specificity of specific networks in task fMRI

**Figure 9:**
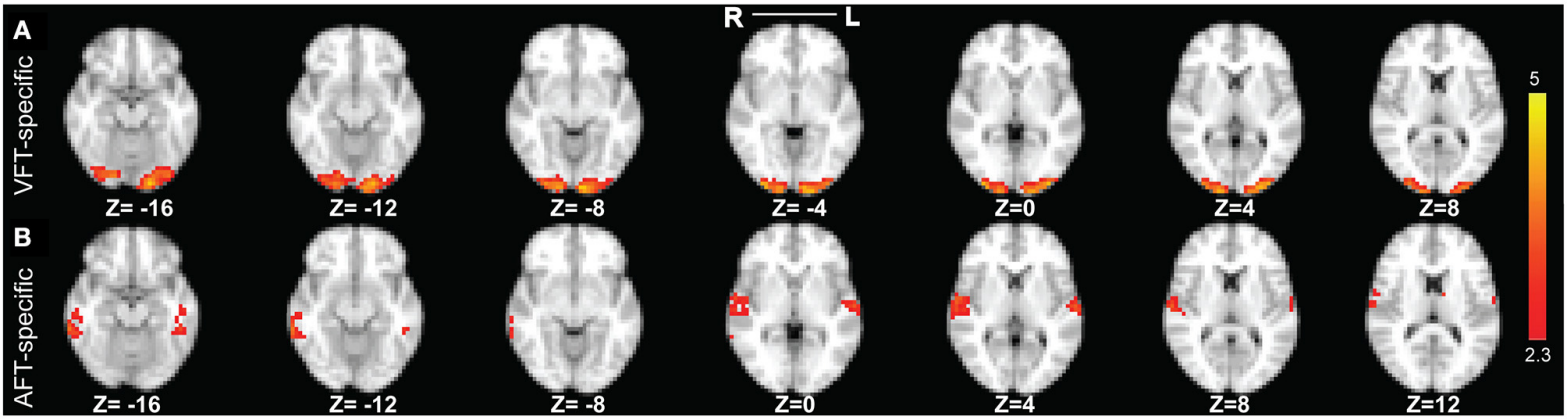
**The two reliable specific networks in finger tapping dataset detected by performing clustering analysis on the results of SSICA**. **(A)** The most reliable network specific to the finger tapping with visual cue condition comprising bilateral occipital pole and occipital fusiform gyri. **(B)** The most reliable network specific to the finger tapping with auditory cue condition comprising bilateral superior temporal gyrus, bilateral Heschl's gyrus, and bilateral middle and inferior temporal gyri (more on the right side). Note that this result is showing the average of spatial maps within each reliable cluster. Z-values range between 2.3 and 5 in both cases.

After running SSICA 75 times (varying notably the maximum number of specific network in each condition from 0 to 3), there were 120 specific components for the finger-tapping with visual-cue and 95 for the finger-tapping with auditory-cue condition. Following the clustering algorithm described in Section Analysis of the Consistency of the Extracted Specific Components in Task fMRI, one significant cluster of components was detected specific to the finger-tapping with visual-cue and one specific to the finger-tapping with auditory-cue condition. These clusters, respectively, included 119 and 94 specific components [as setting the false positive rate (FPR) and the false discovery rate (FDR) thresholds of the clustering analysis at 10%, as recommended by Hyvarinen and Ramkumar (2013) in the context of real fMRI, resulted in one component in each condition not being included into any significant cluster]. The two reliable specific networks, corresponding to the two significant clusters, are illustrated in Figures 9A,B. As explained in Section Analysis of the Specificity of the Extracted Specific Networks in Task fMRI, all the components within each cluster were averaged to obtain the representation of that cluster (the reliable specific network).

Our result in Figure 9A demonstrates that the most reliable visual network comprises bilateral occipital pole and occipital fusiform gyri. Results in Figure 9B demonstrate that the most reliable auditory network comprises bilateral superior temporal gyrus, bilateral Heschl's gyrus, and bilateral middle and inferior temporal gyri (more on the right side). For the cases where 40 or 50 components were extracted at the group level, we found very similar results as when 30 components were extracted.

**Figure 10:**
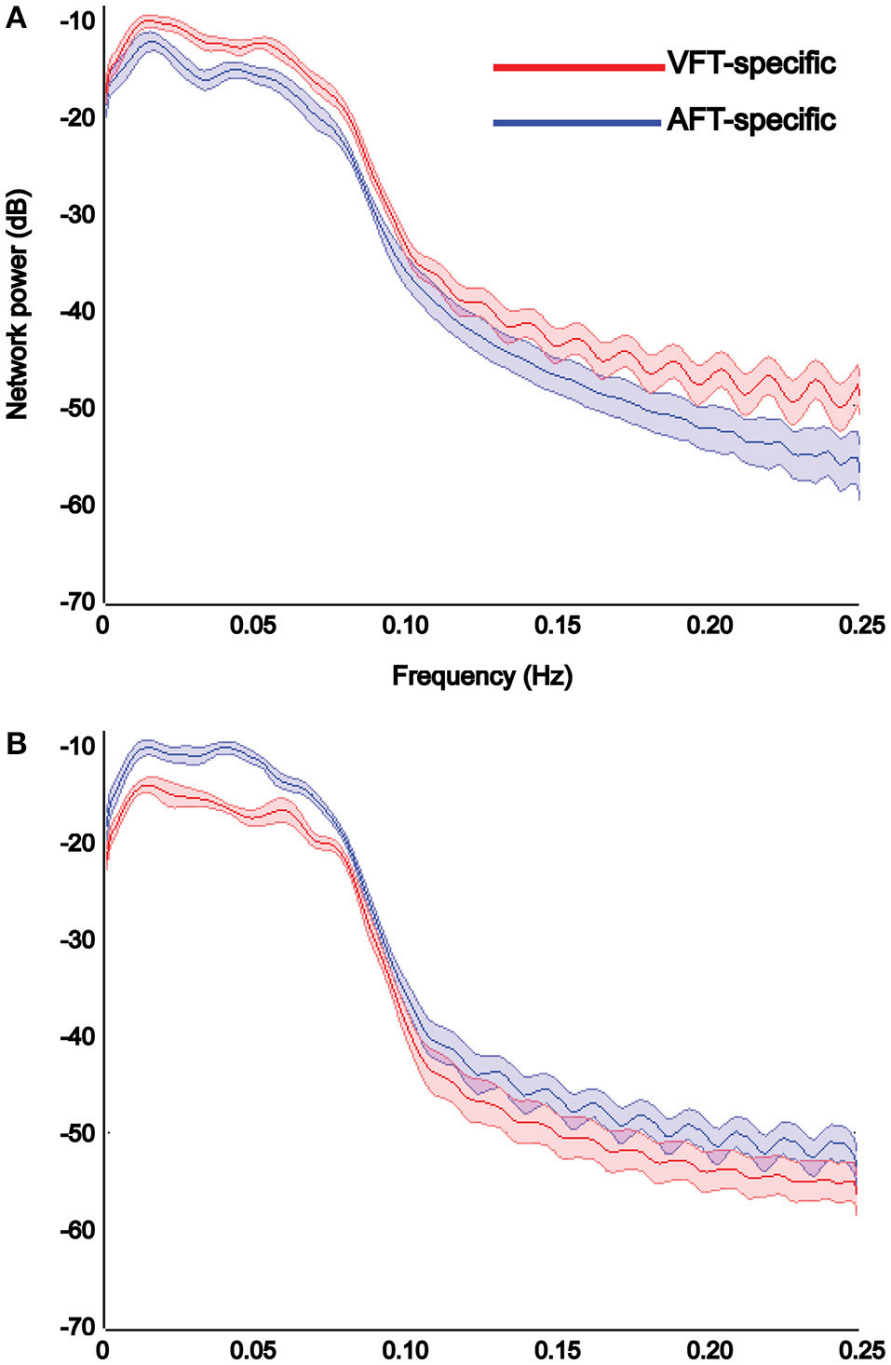
**Results of power spectrum analysis on the temporal dynamics of each detected specific network reported in Figure 9**. The reliable visual network shows increase in the strength of functional connectivity in the finger tapping with visual cue condition compared to the finger tapping with auditory cue condition **(A)**, whereas the reliable auditory network shows the opposite **(B)**. We chose to illustrate the visual networks in red and the auditory network in blue. *X*-axis shows the frequency in Hertz and *Y*-axis indicates the power in decibel. Shaded area shows standard error of the mean (over all subjects).

Results of power spectrum analysis on the temporal dynamics of the detected specific networks (visual and auditory) show that the extracted visual network shows increased functional connectivity, estimated by the power of its corresponding time-course in the [0–0.25 Hz] frequency band, in the finger-tapping with visual-cue condition compared to the finger-tapping with auditory-cue one (Figure 10A), whereas the auditory network shows increased functional connectivity in finger-tapping with auditory-cue condition compared to the finger-tapping with visual-cue one (Figure 10B).

## Discussion

We proposed SSICA (Vahdat et al., 2012; Maneshi et al., 2014) as a new promising method to handle group and/or condition comparison in resting-state functional connectivity studies, built upon the framework of constrained ICA. In the present study, we further evaluated the performance of SSICA using hybrid and real fMRI datasets, and we also modified the initialization of this iterative algorithm to deal with the high-dimensional data of fMRI. We also extended the back-reconstruction method in order to estimate the contribution of every single subject to a component, thus allowing us to generate group-level *t*-statistics maps based on each component extracted using SSICA. We showed that SSICA outperformed the regular gICA approach in extraction accuracy and classification of components. We also demonstrated that SSICA was robust with respect to the number of allowable extracted specific components. When fewer than the actual number of specific components was allowed for extraction, SSICA most often extracted as specific the components with greater power difference across groups. When more specific components than the actual number of specific components were specified, the groups-differences map was either repeated or split over different specific components, depending on the degree of coherence across isolated blobs of the groups-differences map.

Another important property of SSICA is its ability to distinguish structural changes in networks spatial maps (extracted as specific component) vs. changes in the power of spatially identical networks (extracted as shared component). In Vahdat et al. (2012), it was shown that the sensitivity of SSICA was greater compared to regular gICA to detect changes in the power of shared networks across groups as it dissociates structural and temporal differences of the two networks using different cost functions. Here, we systematically showed that in the case of partially-specific network (a network present in both groups but significantly weaker in one) which is a likely scenario in fMRI network analysis, SSICA extracted it as specific if the network's SNR in the weaker group did not exceed half of the background BOLD signal SNR during resting-state conditions; Otherwise this network was extracted and classified as shared. In fact, in this condition, SSICA uses the information from all individuals to better estimate components with significant power in both groups.

SSICA is an exploratory method which, independently of the involved structures, extracts, and classifies components in those common between groups (conditions) and those specific to one group (condition). However, SSICA requires as input the number of networks specific to each group (condition) but the true value of this number is not known a priori. In this context, we proposed in Maneshi et al. (2014), a methodology consisting of launching SSICA multiple times with different numbers of specific components followed by a clustering analysis, to provide us with complementary statistical information regarding the reliability and specificity of the extracted shared and specific networks.

The lack of sensitivity of the regular gICA in detecting the pattern of differences between conditions may be attributed to the fact that in our fMRI task design we did not impose any block timing constraint, to mimic, as much as possible, the resting-state conditions. The validation of group ICA methods is usually done using block-design task-based paradigms, in which BOLD signal variability is mainly due to the context-dependent increased activity within all activated brain areas, rather than intrinsic changes in functional connectivity between areas. Psychophysiological interaction analysis (Friston et al., 1997) is one way to study changes in functional connectivity between different experimental contexts above the main effect of the task. Here, we intentionally separated periods of task and rest to investigate changes in functional connectivity that cannot be explained by timing-related fluctuations. To minimize the effects of habituation, and hence, degradation in task-related BOLD response (Condon et al., 1997), we conducted two runs for each of AFT and VFT conditions. Note that, we could still extract the functionally-related networks using standard GLM analysis in this task design. Therefore, both GIFT and SSICA used information in the temporally unconstrained BOLD signal to detect changes associated to these shared and specific networks.

Several methods have been proposed to perform group ICA in the context of fMRI (Lukic et al., 2002; Guo and Pagnoni, 2008; Calhoun et al., 2009). Some provide a systematic way to obtain single-group statistical maps from individual ICs by combining ICA and clustering methods or canonical correlation analysis (Esposito et al., 2005; Varoquaux et al., 2010; Boly et al., 2012; Hyvarinen and Ramkumar, 2013). However, their extension to perform between-group comparisons remains to be explored. In another gICA method (Lukic et al., 2002), multi-group fMRI data were decomposed into shared and specific components similar to the one used in SSICA; however, the iterative algorithm proposed by Lukic et al. (2002) extracts ICs in separate ICA models for each group and then averages them across groups. Also, in contrast to SSICA that preferentially optimizes the mixing matrix for the extraction of specific components, this algorithm is based on selecting eigenvectors that fulfill a given constraint, which can potentially be obscured by noise. Also, with respect to the unified ICA framework proposed by Guo and Pagnoni (2008), the group data structure of SSICA can be attributed to a class of methods with the most general group ICA model, with no restrictions on the relationship between the subjects' mixing matrices.

Others require extraction of some features from the BOLD signal or sharing a similar time course across subjects as in task-based designs, and hence there is no direct way to apply them in resting-state paradigms (Beckmann and Smith, 2005; Sui et al., 2009a). Compared to these methods of group ICA, SSICA provides a systematic way to simultaneously combine the processes of maximizing independence between the extracted components and performing group comparison in a single step (Vahdat et al., 2012). Also, contrary to feature-extracting methods, SSICA can be directly applied to the resting-state datasets to extract group-specific fMRI networks.

We have previously tested and validated the application of SSICA in real resting-state fMRI data (Maneshi et al., 2014), in which we studied group-specific differences in resting-state networks between patients with unilateral mesial temporal lobe epilepsy (MTLE) and healthy control subjects. We showed that SSICA findings complement results from seed-based analysis and provide more information about the underlying processes resulting in changes of functional connectivity. However, as these two methods measure different aspects of brain activity organization, they may sometimes give apparently inconsistent results. The current study as well as the previous one well demonstrates that SSICA has an advantage over the seed-based method in that an a priori hypothesis is not required and therefore differences between groups can be assessed independently of the involved structures. However, in real case scenarios of fMRI analysis, SSICA needs to be combined with complementary methods such as clustering to find the most reliable or the most stable specific networks among all the extracted ones. Moreover, our current study along with Maneshi et al. (2014) confirm that “reliable” specific networks of each group (or condition) may represent networks with higher functional connectivity (defined by power analysis) in that group (or condition) compared to the other group.

SSICA does not provide *t*-statistics maps for extracted components *per-se*, and it needs to be combined with other methods for assessing consistency of extracted networks across subjects. Several methods such as the back-reconstruction (Calhoun et al., 2001), dual regression (Filippini et al., 2009), and clustering-based approach (Esposito et al., 2005; Hyvarinen and Ramkumar, 2013) have been proposed for generating statistical maps based on the results of group ICA. As explained before, as we were trying to compare SSICA with GIFT method in this study, we chose to adapt and modify the back-reconstruction approach originally proposed for GIFT.

We compared our results with the standard gICA method followed by the back-reconstruction as implemented in GIFT, because we could select the same ICA approach (FastICA) with similar parameters as the one used in SSICA. This provided a fair comparison between the two approaches. In this paper we extended the back-reconstruction method to be combined with the SSICA results. It would be interesting to combine the SSICA results with the dual regression approach; however that was beyond the scope of the current study. In theory, SSICA can be combined with any method of multi-subject group ICA, which provides a measure of consistency across subjects. As the projection of SSICA's specific components on the subspace spanned by the opposite group's data is insignificant (due to the SSICA cost function), performing either one-sample *t*-statistics on the matching group or two-sample *t*-statics between groups results in very similar *t*-statistics maps.

One limitation of SSICA and, in general, concatenation group ICA approaches, is that they require an accurate transformation from subject's functional space to the standard space. Methods such as local linear discriminant analysis (McKeown et al., 2007) and canonical ICA (Varoquaux et al., 2010) are specifically useful to compensate for inter-subject differences when grouping the dataset. Also, it is noteworthy that recent work using SimTB software (Erhardt et al., 2012) has shown that time-concatenation based methods are more robust than thought before with respect to the motion artifacts and variability in the location of components across subjects. Recent work (Himberg et al., 2004; Hyvarinen and Ramkumar, 2013) using clustering approaches to examine the reproducibility of the extracted networks seems a promising method to apply to the results of SSICA's specific networks extracted from multiple runs, with different parameters such as the number of allowable specific components, total number of extracted components, and using different initializations.

## Conclusion

We demonstrated that SSICA is more sensitive than the standard gICA approach to detect patterns of differences in functional connectivity across groups/conditions, particularly in model-free designs such as resting-state fMRI. Furthermore, we showed that SSICA is robust when the number of specific components is mis-specified a priori, and when the orthogonality assumptions between groups are not completely met. We also explained that although our method of functional connectivity analysis, SSICA, and the conventional GLM analysis measure different aspects of brain activity organization, they may complement each other and provide more information about the underlying processes resulting in changes of functional connectivity. More importantly, by combining SSICA with clustering analysis, not only could we validate results obtained from GLM analysis, but we could also detect those changes in functional connectivity across groups which are more reproducible and reliable and should be considered for further inspection.

## Author contributions

Study conception and design: MM; Acquisition of data: MM; Analysis and interpretation of data: MM, SV, JG, CG; Drafting of manuscript: MM; Critical revision: MM, SV, JG, CG.

## Funding

We would like to thank the Canadian institutes of health research (CIHR) that funded our research (grant MOP-130442).

### Conflict of interest statement

The authors declare that the research was conducted in the absence of any commercial or financial relationships that could be construed as a potential conflict of interest.

## Supplementary material

Supplementary Figure 1 **Common resting-state networks extracted by SSICA and gICA**. In the hybrid fMRI data set, using SSICA and the regular gICA approach, four highly-reproducible resting-state networks (default mode, visual, motor, and auditory) were extracted as shared components. As shown, both methods produced highly comparable connectivity maps related to common resting-state networks. Color-coded maps represent one-sample *t*-statistics.
