# Supplementary figures and images for: Validation of Shared and Specific Independent Component Analysis (SSICA) for Between-Group Comparisons in fMRI
Source: Front Neurosci. 2016 Sep 27;10:417. doi: 10.3389/fnins.2016.00417 (PMC5037228; doi:10.3389/fnins.2016.00417)

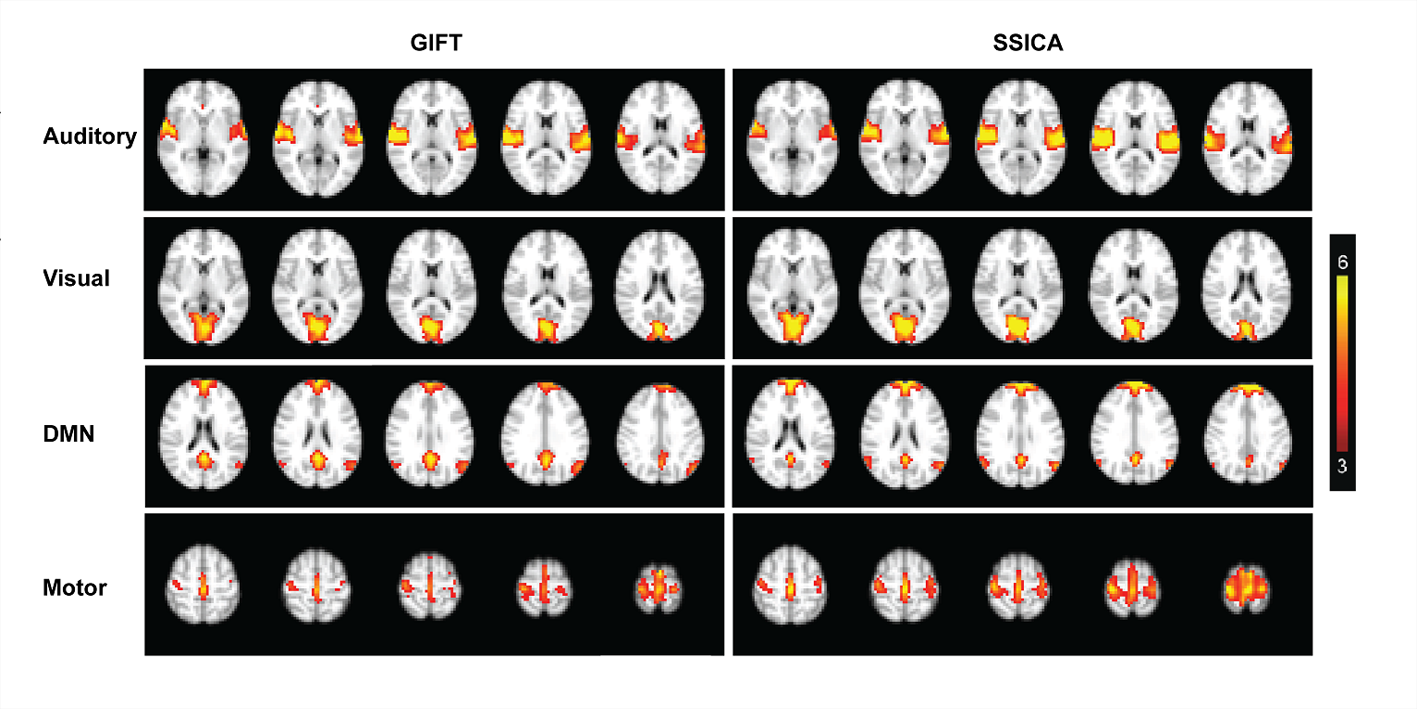

Supplement: Supplementary Figure 1 — Common resting-state networks extracted by SSICA and gICA. In the hybrid fMRI data set, using SSICA and the regular gICA approach, four highly-reproducible resting-state networks (default mode, visual, motor, and auditory) were extracted as shared components. As shown, both methods produced highly comparable connectivity maps related to common resting-state networks. Color-coded maps represent one-sample t-statistics. [file Image1.TIF]
