# Supplementary material for: Validation of Shared and Specific Independent Component Analysis (SSICA) for Between-Group Comparisons in fMRI
Source: Front Neurosci. 2016 Sep 27;10:417. doi: 10.3389/fnins.2016.00417 (PMC5037228; doi:10.3389/fnins.2016.00417)
Supplement: Supplementary file 2 [file Presentation1.pdf]

Appendix A. Standard back-reconstruction within gICA framework

In regular gICA, back-reconstruction is done by projecting the extracted group-level spatial maps onto the subject-level space:

$$\hat{S}_i^j = (\widetilde{D}_i^j A)^+ \, F_i^j \, Y_i^j \quad ; \; i = 1,..,n_j; \; j = 1,2 \qquad (\mathrm{A.1})$$

Where $\hat{S}_i^j$ is the estimated source (spatial map) for subject i in group j, $(.)^+$ denotes the pseudo-inverse operator, and $\widetilde{D}_i^j$ is the sub-matrix corresponding to subject i in group j from row-wise partitioning of the group level de-whitening matrix $(\tilde{G})^+$:

$$(\tilde{G})^+ = \begin{bmatrix} \widetilde{D}_1^1 \\ \vdots \\ \widetilde{D}_{n_1}^1 \\ \widetilde{D}_1^2 \\ \vdots \\ \widetilde{D}_{n_2}^2 \end{bmatrix}$$

Appendix B. Back-reconstruction within SSICA framework

The three-level data reduction in SSICA is summarized as follows:

$$X_i^j = F_i^j \, Y_i^j, \quad i = 1,..,n_j \;, \; j = 1,2 \quad \text{(step 1; subject level)} \qquad (\mathrm{B.1})$$

$$X^j = H^j \begin{bmatrix} X_1^j \\ \vdots \\ X_{n_j}^j \end{bmatrix}, \qquad \text{(step 2; within-group level)} \qquad (\mathrm{B.2})$$

$$X = G \begin{bmatrix} X^1 \\ X^2 \end{bmatrix} = \hat{A} \, \hat{S}, \qquad \text{(step 3; between-group level)} \qquad (\mathrm{B.3})$$

Where $\hat{A}$ and $\hat{S}$, respectively, are the estimated mixing and source matrices resulting from SSICA.

Substitution of Equations B.1 and B.2 into Equation B.3, and simple algebraic factorizations

result in:

$$G^{+}\hat{A}\hat{S}=\begin{bmatrix}H^1\begin{bmatrix}X_1^1\\ \vdots\\ X_{n_1}^1\end{bmatrix}\\ H^2\begin{bmatrix}X_1^2\\ \vdots\\ X_{n_2}^2\end{bmatrix}\end{bmatrix}=\begin{bmatrix}H^1 & 0\\ 0 & H^2\end{bmatrix}\begin{bmatrix}X_1^1\\ \vdots\\ X_{n_1}^1\\ X_1^2\\ \vdots\\ X_{n_2}^2\end{bmatrix}\rightarrow\begin{bmatrix}(H^1)^+ & 0\\ 0 & (H^2)^+\end{bmatrix}G^{+}\hat{A}\hat{S}=\begin{bmatrix}F_1^1\,Y_1^1\\ \vdots\\ F_{n_1}^1\,Y_{n_1}^1\\ F_1^2\,Y_1^2\\ \vdots\\ F_{n_2}^2\,Y_{n_2}^2\end{bmatrix},\quad (\mathrm{B.4})$$

We can split the de-whitening matrix G^{+} into two sub-matrices: $G^{+}\triangleq\begin{bmatrix}D^1\\ D^2\end{bmatrix}$, where D^1 and D^2 are $\mathrm{N}_{g1}\times\mathrm{N}$ and $\mathrm{N}_{g2}\times\mathrm{N}$, respectively. Substituting this in Equation B.4, we get:

$$\begin{bmatrix}(H^1)^+D^1\\ (H^2)^+D^2\end{bmatrix}\hat{A}\hat{S}=\begin{bmatrix}F_1^1\,Y_1^1\\ \vdots\\ F_{n_1}^1\,Y_{n_1}^1\\ F_1^2\,Y_1^2\\ \vdots\\ F_{n_2}^2\,Y_{n_2}^2\end{bmatrix}.\quad (\mathrm{B.5})$$

We can also split the de-whitening matrix $(H^1)^+$ into n_1 sub-matrices B_i^1 $(\mathrm{T}_1\times\mathrm{N}_{g1})$, and $(H^2)^+$ into n_2 sub-matrices B_i^2 $(\mathrm{T}_2\times\mathrm{N}_{g2})$:

$$(H^1)^+\triangleq\begin{bmatrix}B_1^1\\ \vdots\\ B_{n_1}^1\end{bmatrix},\ (H^2)^+\triangleq\begin{bmatrix}B_1^2\\ \vdots\\ B_{n_2}^2\end{bmatrix}.\quad (\mathrm{B.6})$$

By replacing the above equations in the left side of Equation B.5, we get the following set of equations for each subject in group-1 (B.7) and group-2 (B.8):

$$B_i^1D^1\hat{A}\hat{S}=F_i^1\,Y_i^1,\quad i=1,..,n_1,\quad (\mathrm{B.7})$$

$$B_i^2D^2\hat{A}\hat{S}=F_i^2\,Y_i^2,\quad i=1,..,n_2,\quad (\mathrm{B.8})$$

This will result in the back-reconstruction formula for the SSICA algorithm:

$$\hat{S}_i^j=(B_i^jD^j\hat{A})^+F_i^j\,Y_i^j;\quad i=1,..,n_j, j=1,2\quad (\mathrm{B.9})$$
